# Light regulates stomatal development by modulating paracrine signaling from inner tissues

**DOI:** 10.1038/s41467-021-23728-2

**Published:** 2021-06-07

**Authors:** Shenqi Wang, Zimin Zhou, Rini Rahiman, Grace Sheen Yee Lee, Yuan Kai Yeo, Xin Yang, On Sun Lau

**Affiliations:** 1 Department of Biological Sciences, National University of Singapore, Singapore, 117557 Singapore; 2 Present Address: Department of Molecular, Cellular and Developmental Biology, Yale University, New Haven, CT USA

**Keywords:** Developmental biology, Light responses, Plant development, Stomata

## Abstract

Developmental outcomes are shaped by the interplay between intrinsic and external factors. The production of stomata—essential pores for gas exchange in plants—is extremely plastic and offers an excellent system to study this interplay at the cell lineage level. For plants, light is a key external cue, and it promotes stomatal development and the accumulation of the master stomatal regulator SPEECHLESS (SPCH). However, how light signals are relayed to influence SPCH remains unknown. Here, we show that the light-regulated transcription factor ELONGATED HYPOCOTYL 5 (HY5), a critical regulator for photomorphogenic growth, is present in inner mesophyll cells and directly binds and activates *STOMAGEN*. STOMAGEN, the mesophyll-derived secreted peptide, in turn stabilizes SPCH in the epidermis, leading to enhanced stomatal production. Our work identifies a molecular link between light signaling and stomatal development that spans two tissue layers and highlights how an environmental signaling factor may coordinate growth across tissue types.

## Abstract (web summary)

Light promotes stomatal development in plants. Here Wang *et al*. show that light stimulates stomatal development via the HY5 transcription factor which induces expression of STOMAGEN, a mesophyll-derived secreted peptide, that in turn leads to stabilization of a master regulator of stomatal development in the epidermis.

## Introduction

Stomata, the pores on the plant’s epidermis, are key innovations of land plants^1,2^. Through regulating their aperture and number, higher plants gain control over carbon uptake and water usage, allowing adaptation to diverse climates and habitats. In the model plant *Arabidopsis*, the regulation of the number of stomatal guard cells entails developmental control of a specialized epidermal lineage, the stomatal lineage^3,4^. With its responsiveness to a range of external stimuli and accessibility on the leaf surface, the lineage also presents a powerful platform to examine developmental plasticity. The stomatal lineage begins when a meristemoid mother cell (MMC) divides asymmetrically, generating a smaller meristemoid and a larger stomatal lineage ground cell (SLGC). A meristemoid can self-renew but will ultimately differentiate into a guard mother cell (GMC), which divides into a pair of guard cells. In addition to differentiating into a pavement cell, an SLGC can also undergo asymmetric cell division, giving rise to a meristemoid and another SLGC. Thus, the activity of the stomatal precursors, i.e., MMCs and meristemoids, dictates whether the lineage expands or not, and provides flexibility in the production of guard cells and pavement cells on the epidermis.

The specification and proliferation of the stomatal precursors require the master regulator SPEECHLESS (SPCH), a bHLH transcription factor^5,6^. SPCH dimerizes with its bHLH partner, INDUCER OF CBF EXPRESSION 1 (ICE1 or SCREAM/SCRM) or its homolog ICE2/SCRM2, in driving specification and cell divisions^7^. The level of SPCH is regulated by autocrine and paracrine signalings through a repressive signal transduction cascade in the stomatal precursors. The cascade consists of the cell surface receptor complex of ERECTA (ER) family of receptor-like kinases and the receptor-like protein TMM, followed by a MITOGEN-ACTIVATED PROTEIN KINASE (MAPK) module headed by the MAP triple kinase YODA (YDA)^8–11^. Activation of the cascade leads to MPK3/6-mediated phosphorylation and down-regulation of SPCH^12,13^. A stomatal precursor can activate this cascade within itself or in its neighbors through the secreted peptide EPIDERMAL PATTERNING FACTOR2 (EPF2), the ligand for the ER-TMM receptor complex^14–16^. This mechanism helps restrict the activity of the stomatal precursors in ensuring proper patterning. Interestingly, mesophyll cells located internally in the leaf tissue can also influence stomatal precursors through paracrine signaling. STOMAGEN (or EPFL9), another member of the EPF family, is produced by mesophyll cells, but in contrast to EPF2, it promotes SPCH accumulation and stomatal development^17–20^. STOMAGEN interferes with the binding between EPF2 and the ER-TMM receptor, thereby relieving the activity of the repressive MAPK cascade on SPCH^21^.

Light, besides being the energy source for plants, acts as a signal and exerts a major influence on plant development^22^. One notable example is seedling photomorphogenesis, where light-mediated developmental changes occur at cell, tissue, and organ levels to optimize plants for light capture and photosynthesis. The bZIP transcription factor ELONGATED HYPOCOTYL 5 (HY5), a central component in light signal transduction, plays a prominent role in promoting photomorphogenic growth^23,24^. In the dark, it is targeted by the ubiquitin E3 ligase, CONSTITUTIVE PHOTOMORPHOGENIC 1 (COP1), for degradation, and inactivation of COP1 by light-induced photoreceptors leads to the accumulation of HY5 under light^25,26^. In addition, light also induces the transcription of *HY5* and represses the inhibitory phosphorylation on HY5 proteins^27–29^. As a result, HY5 levels are directly proportional to light intensities and correlate with the degrees of photomorphogenic growth^25^. Besides HY5, multiple transcription factors, such as HYH, a HY5 homolog, also contribute to photomorphogenic growth in a partially overlapping manner^26,30^. Like HY5, most of these proteins are targets of COP1.

Light also influences the development of stomata. In the dark, stomatal development is suppressed to conserve water, and light triggers stomatal production for carbon uptake in an intensity-dependent manner and promotes SPCH accumulation^31–33^. Genetic studies suggested that SPCH is inhibited by COP1 through an unknown mechanism that ultimately activates the repressive YDA module^31^. In addition to SPCH, its partner ICE/SCRM proteins are also light-regulated and are directly targeted by COP1 for degradation in the dark^33^. The main role of this regulatory node, however, appears to suppress stomatal development in the absence of light (more in “Discussion”). Thus, how light signals are linked to SPCH and dynamically induce stomatal production remain unresolved.

Here, we identify HY5 as a key player in light-regulated stomatal development. Through genetic and microscopic analyses, we show that *HY5* is required for light responsiveness in stomatal development and impacts the accumulation of SPCH under the light. We also show that *HY5* is co-expressed with *STOMAGEN* in the mesophyll and it directly binds to *STOMAGEN* promoter and induces its expression. We further find that mesophyll-specific HY5 is sufficient to promote stomatal development. Finally, we demonstrate that *STOMAGEN* is important for light-regulated stomatal production and it acts genetically downstream of *HY5* in mediating this response. Based on our data, we conclude that light signals act through HY5 to modulate *STOMAGEN* level, which enables the plasticity of stomatal production toward the light, and this HY5-STOMAGEN module likely represents the missing link that allows light signals to regulate SPCH. Further, since mesophyll cells are the workhorses for photosynthesis, this module may enable these cells in the inner tissue to signal stomatal production on the epidermis for carbon uptake when they are activated by light.

## Results

### *HY5* promotes stomatal production in response to light

**Fig. 1:**
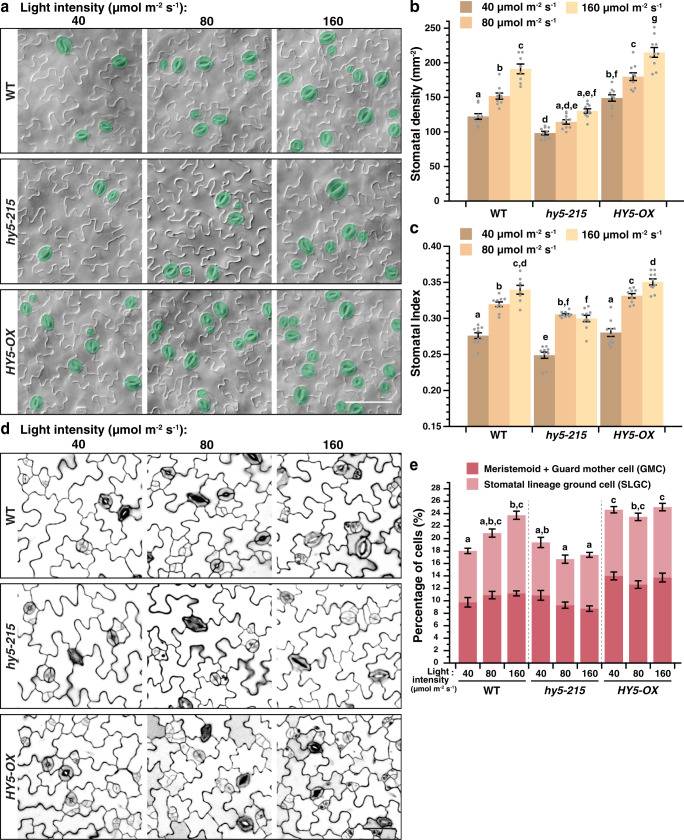
*HY5* is required for the light regulation of stomatal development. **a**–**c** Changes in stomatal production in response to light intensities in WT, *hy5-215*, and *HY5-OX* (overexpression line of *HY5*). Arabidopsis seedlings were grown for 10 days at 22 °C under three distinct light intensities (40, 80, and 160 µmol m^−2^ s^−1^). Representative images (**a**), stomatal densities (**b**), and stomatal indices (**c**; see text) of the abaxial cotyledons are shown. Stomata are pseudo-colored in green (**a**). Scale bar, 80 μm. **d**, **e** Seedlings of the above genotypes (**a**) were grown for 3 days to capture the early cell types of the stomatal lineage under the three light intensities (**a**). Representative confocal images (**d**) and the percentage of these early cell types, which include meristemoids, guard mother cells (GMCs) and stomatal lineage ground cells (SLGCs) (**e**; see text and “Methods”) of the abaxial cotyledons are shown. Scale bar, 40 μm. **b**, **c**, **e** Values are mean + /− SEM, *n* = 10 independent cotyledons. Two-way ANOVA with Tukey’s multiple comparisons test, *P* < 0.05.

To investigate the role of *HY5* in light-regulated stomatal development, we carried out a detailed analysis of stomatal production in *Arabidopsis* with varying *HY5* levels under a series of light intensities. Wild-type (WT), a loss-of-function mutant of *HY5, hy5-215*, and a *HY5* overexpression line, *HY5-OX*^23,34^, were grown under low-, medium-, and high-light intensities (i.e., 40, 80, and 160 µmol m^−2^ s^−1^). Abaxial cotyledons were examined at 10 days post germination (dpg) and their stomatal density (SD, the number of stomata in an area) and stomatal index (SI, the ratio of stomata to all epidermal cells in an area) were scored. In WT, both SD and SI correlate positively with light intensity, i.e., lowest at low light and increased under higher intensities (Fig. 1a–c). Strikingly, the SD of *hy5-*215 was significantly lower than their WT counterpart under all light conditions, while a similar trend was also observed in its SI. In contrast, stomatal production in *HY5-OX* was notably higher than WT. These results suggest a positive role of *HY5* in promoting stomatal development under the light.

As discussed, stomata are derived from the stomatal lineage cells. Thus, factors that influence stomatal production should have a similar impact on the number of the early stomatal lineage cells in developing cotyledons and leaves. To confirm the observed positive effect of *HY5* on stomatal development, we examined the populations of early stomatal lineage cells in WT, *hy5-215*, and *HY5-OX* in younger seedlings (3 dpg) under the three light intensities above. Confocal microscopy was used to image the epidermis of the abaxial cotyledons and the number of the early stomatal lineage cells, i.e., meristemoids, GMCs, and SLGCs, (as well as the other cell types) were counted (see “Methods”). As predicted, in WT, increasing light intensities resulted in an increase in these early stomatal lineage cells (Fig. 1d, e). However, in *hy5-215*, these early lineage populations remained low across light conditions, suggesting a defect in the light-dependent expansion of the lineage. Conversely, the populations of these early lineage cells in *HY5-OX* stayed high under the three light levels. These results provide strong support for the positive role of *HY5* in light-dependent stomatal production and indicate that *HY5* impacts stomatal development early in the lineage.

We also examined the contribution of *HYH*, a homolog of *HY5*^30^, in light-regulated stomatal development. We found that the SD of *hyh-1* was similarly responsive to high-light intensity as its genetic background Ws (Supplementary Fig. 1a). Unexpectedly, the SI of *hyh-1* under this condition was lower than Ws (Supplementary Fig. 1b), suggesting that other non-stomatal cells were over-produced in response to light in the mutant. Nevertheless, stomatal production of the double mutant of *hy5* and *hyh* remained similar to *hy5* (Supplementary Fig. 1a, b). Thus, although the precise nature of *HYH* on cell proliferation remains to be defined, *HY5* likely plays a more prominent role in light-regulated stomatal development. In addition, we investigated the genetic relationship between *HY5* and *COP1* in stomatal development. Consistent with its negative role in light signaling, *cop1-6*^35^ displayed a higher SD compared to WT (Supplementary Fig. 2a, b). Its SI was slightly lower, however, likely due to the overall increase in cell production in the mutant (Supplementary Fig. 2c). Further, a mutation in *HY5* in *cop1-6* did not significantly influence stomatal production. This is not entirely surprising, however, as besides HY5, COP1 directly suppresses a suite of photomorphogenic-promoting transcription factors^26^, and their accumulation in *cop1-6* may have compensated for the loss of *HY5*. As expected, the double mutant of *hyh* and *cop1* also displayed a similar phenotype with *cop1-6* (Supplementary Fig. 1c, d). In sum, our genetic analyses demonstrated that the transcription factor HY5 is critical for the promotion of stomatal development in response to the light.

### *HY5* promotes the light-dependent accumulation of SPCH

**Fig. 2:**
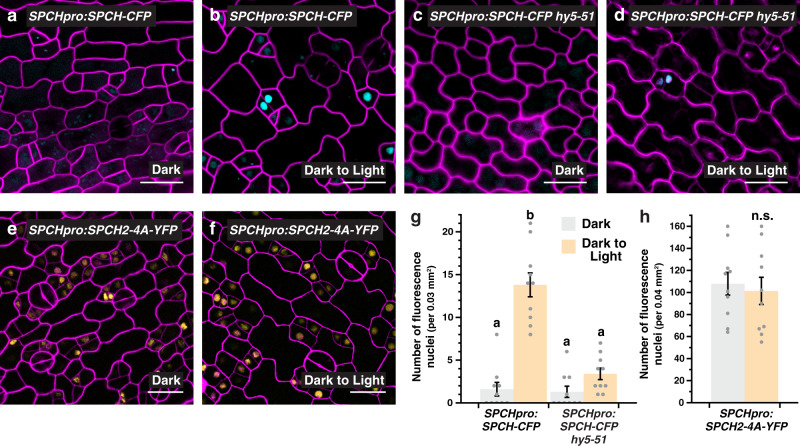
*HY5* regulates the light-induced accumulation of SPEECHLESS. **a**–**d**, **g** Analysis of a translational reporter of *SPEECHLESS* (*SPCH*) in WT (**a**, **b**) and *hy5-51* (**c**, **d**) under light and dark conditions. Confocal images of 3-day-old abaxial cotyledons of *SPCHpro:SPCH-CFP*, which were grown in darkness and were either kept in the dark (**a**, **c**) or transferred to the light for 6 h (**b**, **d**) before imaging. Images were taken with the same excitation and acquisition settings. The numbers of CFP-expressing cells (cyan) in an area are quantified (**g**). **e**, **f**, **h** Analysis of a MAPK-insensitive translational reporter of *SPCH*. Confocal images of 3-day-old abaxial cotyledons of *SPCHpro:SPCH2-4A-YFP* grown in darkness (**e**) or exposed to light (**f**) (see above). The numbers of YFP-expressing cells (yellow) are quantified (**h**; color scheme of bar chart as in **g**). Values are mean + /− SEM, *n* = 10 independent cotyledons. Two-way ANOVA with Tukey’s multiple comparisons test, *P* < 0.01 (**g**) or two-tailed Student’s *t* test, n.s., not significant (**h**). Cell outlines were visualized with propidium iodide (magenta). Scale bar, 20 μm.

We next asked if the light-mediated promotion of stomatal development by *HY5* involves the regulation of *SPCH*. We first examined the effect of light on SPCH accumulation by using a translational reporter of *SPCH* (*SPCHpro:SPCH-CFP*). Similar to a previous report^33^, we observed that *SPCH* expression can be induced by light (Fig. 2a, b, g). The dark-grown reporter line had few numbers of cells with detectable CFP signals, but the numbers were substantially increased when the seedlings were shifted to light for 6 h. To test if *HY5* is involved in the induction, we introgressed *SPCHpro:SPCH-CFP* into *hy5-51*, another null allele of *HY5* that displayed a defect in stomatal response to light (Supplementary Fig. 1a, b), and analyzed its effect on *SPCH* expression. As expected, dark-grown *SPCHpro:SPCH-CFP hy5-51* showed similar low numbers of CFP-expressing cells compared to its counterpart in WT background (Fig. 2a, c, g). However, the light-induced accumulation of *SPCH* was severely compromised in the *hy5* mutant (Fig. 2d, g). We further assayed the expression changes of two gene targets of SPCH during the dark-to-light transition in WT and *hy5-51* (Supplementary Fig. 3). The two genes, *BASL* and *EPF2*, are direct targets of and are activated by SPCH^36^. Correlating with the SPCH level in our confocal assays, our RT-qPCR analyses showed that the expression of the two genes was induced by light in WT but the induction was significantly reduced in the *hy5* mutant. These results indicate a key role of *HY5* in promoting *SPCH* expression under the light.

To further understand the regulatory mode of *SPCH* by light, we investigated if post-translational control of *SPCH* may play a role. As discussed earlier, SPCH proteins are known to be phosphorylated and down-regulated through the repressive MAPK module^12^. Alanine substitution of the critical amino acids targeted by MAPKs on SPCH can stabilize it, resulting in the overaccumulation of SPCH and the stomatal precursors^12^. As expected, our MAPK-insensitive reporter of *SPCH* (*SPCHpro:SPCH2-4A-YFP*) produced many SPCH-expressing cells under light (Fig. 2f). Interestingly, we found that similar numbers of SPCH-positive cells were already present in the dark-grown reporter without light treatment (Fig. 2e, h). The accumulation of SPCH2-4A-YFP, but not SPCH-CFP, in darkness suggests that light can regulate *SPCH* at the protein level. In the dark, SPCH proteins are likely actively suppressed by the MAPKs, and thus, light may promote SPCH expression through inhibiting the repressive MAPK module. In line with the above results, we found that the MAPK-insensitive reporter of *SPCH* behaved similarly in the *hy5* mutant, further suggesting *HY5* may modulate SPCH level through the MAPK signaling (Supplementary Fig. 4).

We also tested the functional dependence of *HY5* on *SPCH* in stomatal development. A double mutant between *HY5-OX* and *spch-3* was generated through the genetic crossing, and the enhanced stomatal number phenotype of *HY5-OX* was completely suppressed in the double mutant (Supplementary Fig. 5). This result confirmed that *HY5* acts upstream of *SPCH*.

### HY5 upregulates the expression of *STOMAGEN*, a mesophyll-derived secreted peptide

Based on these data, we hypothesized that *HY5* promotes SPCH accumulation and stomatal production through suppressing MAPK signaling. Since HY5 is a transcription factor, it most likely influences this process by regulating gene expression. We suspected *STOMAGEN*, which encodes the secreted peptide that stimulates stomatal development, as a prime target under HY5’s influence. This is because *STOMAGEN* acts precisely to inhibit the repressive MAPK module in the stomatal lineage, resulting in the stabilization of SPCH^20,21^. In addition, a previous report has indicated that the expression of *STOMAGEN* can be induced by light^37^.

**Fig. 3:**
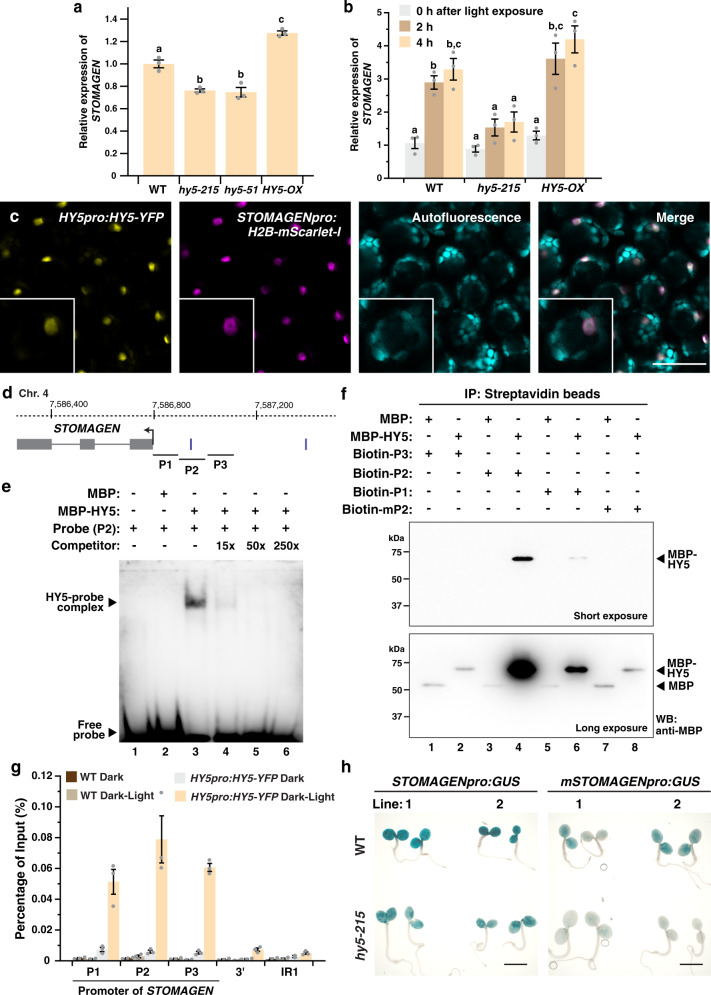
*HY5* directly binds and regulates the expression of *STOMAGEN*. **a**, **b** Gene expression analyses of *STOMAGEN* in WT, *hy5-215*, *hy5-51* (a only), and *HY5-OX* by RT-qPCR. In (**a**), RNA was extracted from 3-day-old seedlings grown under the light. In (**b**), seedlings were grown in darkness for 4 days and were exposed to light for 0, 2, and 4 h before harvest. Values are mean + /− SEM, *n* = 3 biological replicates. One-way (**a**) or two-way (**b**) ANOVA with Tukey’s multiple comparisons test, *P* < 0.01. **c** Co-expression of *HY5* and *STOMAGEN* in the mesophyll layer. Confocal analysis of 3-day-old abaxial cotyledons of a transgenic seedling harboring both *HY5pro:HY5-YFP* and *STOMAGENpro:H2B-mScarlet-I*. From left: YFP signals (yellow), mScarlet-I signals (magenta), autofluorescence (cyan) and merged image of all three channels. Scale bar, 50 μm. Three independent cotyledons were examined with similar results (**d**) Gene structure of *STOMAGEN*. Arrow indicates the translational start site. Vertical bars mark the position of a Z-box (upstream of TSS only). P1 to 3 represent region(s) tested by EMSA (**e**), DNA pull down (**f**) and ChIP-qPCR (**g**). **e** EMSA analysis showing the binding of HY5 to a promoter fragment of *STOMAGEN* (P2). Recombinant MBP (control) and MBP-HY5 were assayed for binding with the biotin-labeled P2 probe. An unlabeled probe (competitor) was used to determine binding specificity (lanes 4–6). **f** DNA pull-down analysis showing the binding of HY5 to P2 and its dependence on the Z-box. Biotin-labeled probes, including a P2 probe with a mutated Z-box (mP2), were used to pull-down recombinant MBP (control) and MBP-HY5. Results were analyzed by western blotting using an anti-MBP antibody. **g** ChIP-qPCR assays were performed on WT and *HY5pro:HY5-YFP* using an anti-GFP antibody. Seedlings were grown for 4 days in darkness before exposed to light for 4 h or kept in the dark. Promoter regions of *STOMAGEN* (see d) were tested. A genomic region downstream of *STOMAGEN* and IR1 (see “Methods”) were used as negative controls. Values are mean + /− SEM, *n* = 3 technical replicates. Assay was repeated with similar results. **h** GUS reporter assay of two independent lines of *STOMAGENpro:GUS* and *mSTOMAGENpro:GUS*, which carries a mutated Z-box in the P2 region, in WT and *hy5-215*. Seedlings were grown for 3 days under the light. Scale bar, 2 mm. Light intensity used: 200 (**a**) or 100 µmol m^−2^ s^−1^ (**b**, **c**, **g**, **h**). The experiments in (**e**) and (**f**) were carried out two times with similar results.

To study the effect of *HY5* on *STOMAGEN* expression, we first examined the level of *STOMAGEN* transcripts in WT, loss-of-function mutants of *hy5*, and *HY5-OX* by RT-qPCR. Compared to WT, the two *hy5* mutants, *hy5-215* and *hy5-51*, had around 20% less *STOMAGEN* transcripts when grown under light (Fig. 3a). In the contrary, *HY5-OX* accumulated 20% more than WT. Consistent with its role in promoting *STOMAGEN* expression, the transcript and protein levels of the native promoter-driven, VENUS-tagged STOMAGEN peptide^17^ were also reduced in *hy5-215* compared to the WT background (Supplementary Fig. 6). To provide insights into the dynamics of *STOMAGEN* regulation by *HY5*, we studied the transcriptional response of *STOMAGEN* during the dark-to-light transition in WT, *hy5-215*, and *HY5-OX* (Fig. 3b). In WT, we observed a robust induction of *STOMAGEN* by light, where its level was increased by around threefold after light exposure for 2 h. In *hy5-215*, however, this induction was substantially muted with mild increases upon light treatment. In *HY5-OX*, expression of *STOMAGEN* was still significantly induced by light and transcripts of *STOMAGEN* accumulated to high levels upon light treatment. The reason *STOMAGEN* in *HY5-OX* remained low at 0 h is likely due to the active elimination of the overexpressed HY5 proteins by COP1 in darkness^25^. In addition, to examine the effect of *HY5* on *STOMAGEN* further, we generated stable *STOMAGEN* promoter-driven *GUS* reporter lines (*STOMAGENpro:GUS*) in WT and *hy5* mutant backgrounds. In line with the above results, our histochemical staining and RT-qPCR analyses showed that *GUS* expression was induced by light in WT but the induction was compromised in *hy5-215* (Supplementary Fig. 7). These expression analyses suggest that *HY5* positively regulates *STOMAGEN* and is involved in its responsiveness to light.

### *HY5* is expressed in the mesophyll and directly binds to *STOMAGEN* in a light-dependent manner

To dissect how *HY5* influences *STOMAGEN* expression, we next asked if the expression domain of *HY5* overlaps with that of *STOMAGEN*. *STOMAGEN* is expressed in the mesophyll cells in immature cotyledons and leaves^17,19^. On the other hand, *HY5* is known to be broadly expressed in plants^23,38^, but it is unclear if *HY5* is specifically expressed in the mesophyll layer. To this end, we employed a translational reporter of *HY5*, *HY5pro:HY5-YFP*, and examined the expression of *HY5-YFP* in the mesophyll. Our confocal analyses targeted to the inner tissues detected positive YFP signals (yellow) in the mesophyll cells of young cotyledons but not in the WT control (Supplementary Fig. 8a, b). To further confirm their co-expression, we generated a transcriptional reporter of *STOMAGEN*, *STOMAGENpro:H2B-mScarlet-I*, and crossed it into *HY5pro:HY5-YFP*. In the mesophyll layer of plants expressing both constructs, we found that the expression domain of *HY5* and *STOMAGEN* largely overlaps (Fig. 3c). Thus, the reporter analyses indicate that HY5 proteins are present in the *STOMAGEN*-expressing mesophylls, and suggest HY5 may regulate *STOMAGEN* within these cells.

Since HY5 is a bZIP transcription factor, the confocal results led us to further test if HY5 can directly regulate *STOMAGEN*. HY5 recognizes the ACGT-containing elements, which include G-box (cACGTg), Z-box (or G/A box, e.g., tACGTg), and their variants^39,40^. Intriguingly, a Z-box motif is present within the first 500 bp of the *STOMAGEN* promoter (Fig. 3d). We first tested the potential direct association between HY5 and the *STOMAGEN* promoter in vitro by Electrophoretic Mobility Shift Assay (EMSA). To generate the probe, the Z-box-containing region of the *STOMAGEN* promoter (P2, Fig. 3d) was synthesized and labeled with biotin. Recombinant HY5 proteins, tagged with maltose-binding protein (MBP), and MBP alone were purified from *E. coli* (Supplementary Fig. 9a) and incubated with the probe. The presence of MBP-HY5, but not MBP, resulted in a shift in the mobility of the labeled probe (Fig. 3e; lane 3 vs. 2). Further, the addition of excess unlabeled probes (i.e., “Competitor”) reduced the intensities of the shifted band in a concentration-dependent manner (Fig. 3e, lanes 4–6). In addition, competitors derived from P1 or P3 did not effectively compete against the P2 probe (Supplementary Fig. 9b). Thus, the EMSA assay showed that HY5 can physically interact with the *STOMAGEN* promoter in vitro. To strengthen the EMSA results and test the importance of the Z-box for the binding of HY5, we performed in vitro DNA pull-down assays using biotin-labeled DNA probes from P1 to P3 and a Z-box-disrupted P2 region (mP2) against the recombinant MBP-HY5 (Fig. 3f). We found that the P2 region can pull-down MBP-HY5, but not MBP, effectively (Fig. 3f, lane 4 vs. 3), and mutations in the Z-box (mP2) abolished its interaction with HY5 (Fig. 3f, lane 4 vs. 8,). We also found that HY5 has a weak but detectable affinity to the P1 region in the assay (Fig. 3f, lane 6).

In a genome-wide study of HY5-binding sites, *STOMAGEN* was among the 3000 potential direct targets of HY5^39^. To confirm the direct association between HY5 and the *STOMAGEN* gene in vivo, we performed detailed chromatin immunoprecipitation (ChIP) assays using our established protocol that can detect cell/tissue-specific protein–DNA interactions^36,41^. WT and the transgenic line expressing YFP-tagged HY5 natively (*HY5pro:HY5-YFP*) were grown in the darkness and were either left further in the dark or exposed to light for 4 h before harvest. The light treatment serves to promote the accumulation of HY5 whereas dark-grown plants contained minimal HY5 proteins (Supplementary Fig. 9c). In light-treated *HY5-YFP*, we observed substantial enrichment of the genomic regions corresponding to the *STOMAGEN* promoter (P1 to P3), but not the control regions (3’ and IR1, see “Methods”) (Fig. 3g). Notably, these enrichments are dependent on both light and HY5, as the promoter regions were not enriched in dark-grown *HY5-YFP* and all of the WT samples.

We also tested the importance of the HY5-binding Z-box on *STOMAGEN* expression in vivo. We constructed the Z-box-mutated version of our *GUS* reporter of *STOMAGEN* (*mSTOMAGENpro:GUS*) in WT and *hy5* mutant background. During dark-to-light transition, like *STOMAGENpro:GUS* in *hy5* described above, the induction of *GUS* expression by light in the mutated reporter lines was not observed, suggesting that the Z-box is important for light-dependent *STOMAGEN* expression (Supplementary Fig. 7). We further examined the *GUS* reporters under standard light conditions (Fig. 3h). Similar to the RT-qPCR results (Fig. 3a), the activity of *STOMAGENpro:GUS* was lower in *hy5-215* compared with WT under light. In WT, the activity of *mSTOMAGENpro:GUS* was also lower than *STOMAGENpro:GUS*, which again supported the importance of the Z-box in *STOMAGEN* expression. Interestingly, we found that the activity of *mSTOMAGENpro:GUS* was further reduced in *hy5*. This suggests that, in addition to the Z-box at P2, HY5 may still be able to regulate *STOMAGEN*, perhaps through binding to the other regions of its promoter (such as P1). Overall, our results suggest that light-induced HY5 can accumulate in the mesophyll layer and directly binds and activates *STOMAGEN*.

### Mesophyll-derived HY5 is sufficient in promoting light-induced stomatal development

As mentioned above, *HY5* is broadly expressed in plants^23,38^. Besides its expression in the mesophyll (Fig. 3c), we found that *HY5* is also expressed in the epidermis, including the stomatal lineage (Supplementary Fig. 8c). This raises a scenario where both the mesophyll- and epidermal-derived HY5 may regulate stomatal production. Since our results so far suggest a role of HY5 in the mesophyll cells through activating *STOMAGEN*, we specifically tested the role of the mesophyll-derived HY5 on light-dependent stomatal development.

**Fig. 4:**
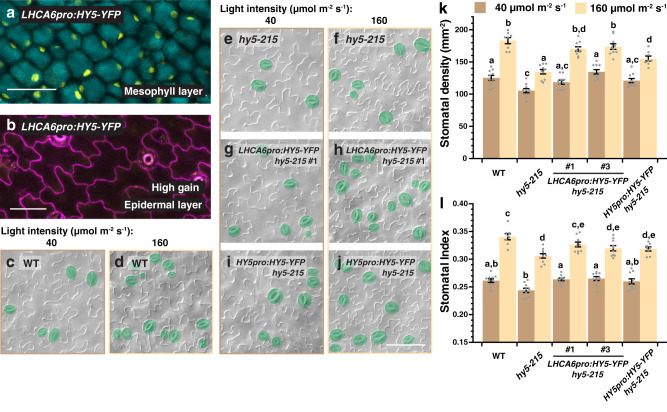
Mesophyll-derived HY5 is capable of driving stomatal development. **a**, **b** Confirmation of the mesophyll-specific expression of *LHCA6pro:HY5-YFP*. Confocal analysis of 3-day-old abaxial cotyledons of *LHCA6pro:HY5-YFP* in the mesophyll layer (**a**) and the epidermis (**b**). YFP signals (yellow) were detected in (**a**) but not in (**b**). Autofluorescence (cyan) of mesophyll cells was captured in (**a**). In (**b**), cell outlines were visualized with propidium iodide (magenta). Scale bar, 50 μm. Three independent cotyledons were examined with similar results. **c**–**l** Genetic complementation of stomatal defects of *hy5* by *LHCA6pro:HY5-YFP*. Arabidopsis seedlings of WT, *hy5-215*, two independent lines of *LHCA6pro:HY5-YFP* in *hy5-215* and *HY5pro:HY5-YFP* in *hy5-215* were grown for 10 days at 22 °C under low- and high-light intensities (40 and 160 µmol m^−2^ s^−1^). Representative images (**c**–**j**), stomatal densities (**k**), and stomatal indices (**l**) of the abaxial cotyledons are shown. Stomata are pseudo-colored in green (**c**–**j**). Scale bar, 80 μm. Values are mean + /− SEM, *n* = 10 independent cotyledons. Two-way ANOVA with Tukey’s multiple comparisons test, *P* < 0.05.

To this end, we generated a mesophyll-specific construct of *HY5*, using the mesophyll-specific *LHCA6* promoter^42^, and transformed it into the *hy5-215* mutant to test for complementation of the stomatal defect. We first confirmed the tissue specificity of the *LHCA6pro:HY5-YFP* line through confocal microscopy. Indeed, YFP signals were only observed in the mesophyll layer but not on the epidermis (Fig. 4a, b). Next, light-dependent stomatal production of two independent lines of *LHCA6pro:HY5-YFP* in *hy5-215*, as well as WT, *hy5-215*, and *HY5pro:HY5-YFP hy5-215*, were examined under low and high-light intensities (i.e., 40 and 160 µmol m^−2^ s^−1^) (Fig. 4c–l). Similar to our results above, the SD and SI of WT responded positively to light, while the values of these stomatal indicators were generally lower in *hy5-215* (Fig. 4c–f, k, l). Interestingly, we found that, similar to *HY5pro:HY5-YFP*, the two *LHCA6pro:HY5-YFP* lines largely rescued the stomatal defects of the *hy5* mutant, restoring stomatal production to a level similar to WT under both low- and high-light conditions (Fig. 4g–j, k, l). Although we cannot exclude the role of epidermal-derived HY5 on stomatal development, these results strongly suggest that mesophyll-derived HY5 is sufficient in promoting stomatal development, and likely plays a substantial role in mediating the light-dependent stomatal response.

### *STOMAGEN* is important for the HY5-mediated promotion of stomatal development

**Fig. 5:**
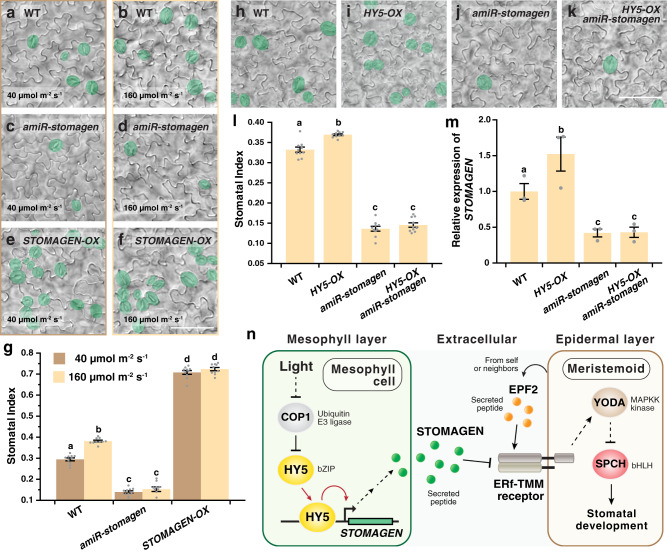
*STOMAGEN* is important for the *HY5*-mediated promotion of stomatal development. **a**–**g** Light-induced stomatal development is impaired in mutants of *STOMAGEN*. Arabidopsis seedlings of WT, *amiR-stomagen* (knockdown line of *STOMAGEN*), and the overexpressor of *STOMAGEN* (*STOMAGEN-OX*) were grown for 10 days under either low (40 µmol m^−2^ s^−1^) or high (160 µmol m^−2^ s^−1^) light intensity. Representative images (**a**–**f**) and stomatal indices (**g**) of the respective abaxial cotyledons are shown. **h**–**l** Suppression of the stomatal overproduction phenotype of *HY5-OX* in a knockdown mutant of *stomagen*. Arabidopsis seedlings of WT, *HY5-OX*, *amiR-stomagen,* and the double-mutant *HY5-OX amiR-stomagen* were grown for 10 days under 100 µmol m^−2^ s^−1^ of light. Representative images (**h**–**k**) and stomatal indices (**l**) of the respective abaxial cotyledons are shown. Stomata are pseudo-colored in green (**a**–**f**, **h**–**k**). Scale bar, 80 μm. (**m**) Gene expression analysis of *STOMAGEN* in WT, *HY5-OX*, *amiR-stomagen* and *HY5-OX amiR-stomagen* by RT-qPCR. RNA was extracted from 3-day-old seedlings grown under 100 µmol m^−2^ s^−1^ of light. Values are mean + /− SEM, *n* = 10 independent cotyledons (**g,** **l**) or three biological replicates (**m**). Two-way (**g**) or one-way (**l**, **m**) ANOVA with Tukey’s multiple comparisons test, *P* < 0.01 (**g**, **l**) and *P* < 0.05 (**m**). **n** Model of the HY5-STOMAGEN module in promoting light-mediated stomatal development. Light suppresses COP1 and leads to HY5 accumulation. In mesophyll, HY5 binds and induces *STOMAGEN*. Increased production of STOMAGEN in turn inhibits the repressive signaling pathway of the epidermal stomatal lineage, leading to the accumulation of SPCH and enhanced stomatal production.

The regulatory role of HY5 on *STOMAGEN* suggests that *STOMAGEN* is involved in the light-mediated promotion of stomatal development and acts downstream of *HY5* in this process. To test this, we first examined the effect of light on stomatal indices in WT, a knockdown mutant of *STOMAGEN* (*amiR-stomagen*)^17^ and an overexpressor of *STOMAGEN*, *STOMAGEN-OX* (see “Methods”). As expected, the knockdown and gain-of-function mutants of *STOMAGEN* displayed a general reduction and enhancement in stomatal production, respectively (Fig. 5a–g and Supplementary Fig. 10a). However, in addition to this defect, both *amiR-stomagen* and *STOMAGEN-OX* also showed reduced responsiveness toward changing light intensity (Fig. 5c–g). Unlike WT, their stomatal index under low- and high light were not significantly different. These results support *STOMAGEN* is critical for the light-regulated process.

We also investigated the genetic relationship between *HY5* and *STOMAGEN* by introgressing *HY5-OX* into *amiR-stomagen*. We found that the enhanced stomatal production phenotype of *HY5-OX* is completely suppressed in the *amiR-stomagen* mutant background, suggesting that *STOMAGEN* is epistatic to *HY5* and that the role of *HY5* in stomatal development may depend on *STOMAGEN* (Fig. 5h–l and Supplementary Fig. 10b). Corresponding to the phenotype, our RT-qPCR analyses also showed that, while *HY5-OX* produced more *STOMAGEN* transcripts than WT, the double-mutant *HY5-OX amiR-stomagen* had a much reduced level, similar to that of *amiR-stomagen* (Fig. 5m). The reduced *STOMAGEN* level likely led to the lowered SI in *HY5-OX amiR-stomagen*. Thus, our analyses support the role of *STOMAGEN* in light-regulated stomatal development and provide genetic evidence that *HY5* functions upstream of *STOMAGEN* in influencing the stomatal production in response to light.

## Discussion

Overall, our work reveals a molecular mechanism that allows plants to modulate SPCH and stomatal production in response to light. Based on our results, we propose a model where light, which inactivates COP1, promotes the accumulation of HY5 (Fig. 5n). The HY5 proteins in the mesophyll then directly bind to the promoter of *STOMAGEN* and induce its expression. In turn, the increased level of the STOMAGEN peptides in the apoplast interferes with the EPF2-ER-TMM interaction and inhibits the YDA-mediated repressive MAPK module in the stomatal precursors. The suppression of the pathway leads to stabilization of SPCH and enhanced stomatal production. Since light intensity determines the level of HY5 (Supplementary Fig. 11)^25^, light can modulate the expression of *STOMAGEN*, and thus SPCH and stomatal number, in a light-dependent manner.

Our results reveal an intriguing mechanism where an environmental stimulus influences cells from one tissue, and through them, gains developmental control over cells from another tissue layer. This regulation suggests that stomatal development in the epidermis is tightly coordinated with the inner mesophyll—at least in light-regulated development. In a plant, mesophyll cells are the major site of photosynthesis and they contain large populations of chloroplasts. Light is essential for chloroplast development and HY5 promotes both the biogenesis of the organelle and the synthesis of chlorophyll by directly activating genes involved in these processes^39,43–45^. Thus, it is tempting to speculate that while light-activated HY5 prepares mesophylls to carry out photosynthesis, it also turns these “primed” mesophylls into signaling centers that activate their neighboring stomatal lineage cells, increasing pore formation for the intake of carbon dioxide for photosynthesis. This mechanism may ensure that the supply of carbon dioxide, which is governed by stomata, meets the demand of the mesophyll cells when they are activated by light. It is also worth noting that, in addition to producing stomata, the stomatal lineage contributes to epidermal and overall leaf growth^46,47^. Thus, the HY5-STOMAGEN module may also function as a signaling node for growth control when light activates the mesophyll cells.

The discovery of the HY5-STOMAGEN module also puts a spotlight on *STOMAGEN* as a regulatory node in controlling stomatal numbers. It has been shown previously that MONOPTEROS (MP)/ARF5, a transcription factor that regulates auxin-responsive genes, can bind to the auxin response elements on *STOMAGEN* and repress its expression, leading to auxin-mediated suppression of stomatal development^48^. Our findings thus further strengthen *STOMAGEN* as a key node, where an external stimulus, i.e., light, and an intrinsic signal may converge on a single gene to coordinate stomatal production. It will be interesting to investigate the interplay between light and auxin in regulating stomatal development and whether this is mediated through competition in binding between MP and HY5 on *STOMAGEN*.

Besides the HY5-STOMAGEN module described here, the ICE/SCRM proteins, i.e., the dimeric partners of SPCH, and COP1 also constitute a regulatory node in light-regulated stomatal development^33^. Why do plants need multiple regulatory pathways in the light response? We propose that the two strategies serve two overlapping yet distinct functions. The COP1-ICE module may act as a binary switch to either restrict or allow stomatal progression in response to dark or light. In the darkness, the module limits the progression of the stomatal lineage by eliminating ICE proteins, which also participate in the subsequent fate transitions in the lineage^7^, and suppresses stomatal formation in the dark. Under the light, ICEs accumulate, but their level becomes saturated even at very low-light intensity^33^, likely rendering ICEs no longer a limiting factor. On the other hand, the HY5-STOMAGEN module, besides coordinating differentiation between tissue layers, fulfills the role of a light-tunable switch in stomatal production. The level of HY5 correlates quantitatively to a range of light intensity and genetic studies also showed that varying *STOMAGEN* level produces a broad range of stomatal density^17,25^. Thus, through the HY5-STOMAGEN module, plants may adjust the extent of stomatal production according to ambient light levels. Therefore, the two pathways combine to offer tight control in darkness and flexibility under light, providing adaptability for plants in diverse light conditions.

In addition to COP1 and HY5, other light signaling components/effectors also play a role in regulating stomatal development, although the underlying mechanism remains unclear^32,49^. One notable factor is PHYTOCHROME-INTERACTING FACTOR 4 (PIF4), which, like COP1, is a repressor of light signaling, but unexpectedly, it acts as a positive factor in light-regulated stomatal development^32,50^. We recently showed that under high temperature, PIF4 does repress stomatal development by directly suppressing *SPCH* expression in the stomatal precursors^51^. Since the expression of *PIF4* is highly induced by high temperature, its mode of action in light response may differ and would require further investigation. Nevertheless, this highlights that many light signaling components have additional roles beyond light response. In fact, the level of HY5 is regulated by low temperatures and heat shock^52,53^. Thus, it would be interesting to test if other external stimuli may influence stomatal development through HY5 and STOMAGEN.

During the revision of our work, a study was published, which surprisingly suggests that *HY5* has minimal influence on stomatal development^54^. The experimental conditions used in the study, however, were quite different from ours, including the stage of the leaves/cotyledons examined, assay methods, growth temperature, humidity, etc. Among these variations, a notable difference is photoperiod: whereas we employed long days (16-h light; 8-h dark) for plant growth, the study used short day (11-h light; 13-h dark) conditions. Since short days have been shown to suppress stomatal production^51,55^, one possible reason for the observed variation may be that this photoperiod renders plants less sensitive to the influence of *HY5*, resulting in a less discernable phenotype. Although the reasons behind this would need further investigation, the discrepancy underscores the highly sensitive and complex nature of stomatal production which integrates multiple environmental signals.

The HY5-STOMAGEN regulatory module also offers a glimpse into when and how environmental signaling connects with developmental processes during evolution. *STOMAGEN* is believed to first appear in early vascular plants, such as lycophytes, in the late Devonian period^17,56^. With the drastic decline in atmospheric CO_2_ level during the time, early vascular plants possessed substantially higher stomatal density than their ancestors^57^ and *STOMAGEN* may be involved in this adaptation. On the other hand, light signaling networks and *HY5* have a more ancient origin and homologs of *HY5* can be found in mosses and liverworts^58,59^. Thus, when early vascular plants continued to evolve, some of these plants may have tapped into the control of stomatal development and connect it with the light signaling pathway. Although exactly when this happened remains unknown, gaining control of this “novel” *STOMAGEN* gene represents a relatively straightforward way to link mesophyll cells with precise stomatal control and enable light-regulated plasticity in stomatal production. It will be interesting to examine if the HY5-STOMAGEN regulatory module is present in other plant lineages and determine when this regulatory mode first appeared.

## Methods

### Plant materials and growth conditions

The *Arabidopsis* ecotype Columbia-0 (Col) was used as the wild-type control in all experiments, except otherwise stated. The following mutants and transgenic lines used in this study were reported previously: *hy5-215*;^23^ *hy5-51* (Salk_096651 or *hy5q*);^60^ *cop1-6*;^35^ *hy5-215 cop1-6*;^61^ *hyh-1*^30^, *HY5-OX* (*UBQpro:HA-HY5*);^34^ *SPCHpro:SPCH-CFP*;^62^ *SPCHpro:SPCH2-4A-YFP*;^12^ *HY5pro:HY5-YFP* in *hy5-1*;^63^ *STOMAGENpro:STOMAGEN-VENUS*; and *amiR-stomagen*^17^. Seedlings were grown on ½ strength Murashige and Skoog (MS) agar media (1%) at 22 **°**C in environmental control chambers (Percival) at the indicated light intensity. Long-day conditions (16-h light/8-h dark) were used in all experiments. The hypocotyl phenotypes of *hy5-215* and *HY5-OX* can be readily observed (Supplementary Fig. 12).

### Accession numbers

Sequence information of the genes studied in this article can be obtained from The Arabidopsis Information Resource (TAIR) (http://www.arabidopsis.org) with the following accession numbers: *HY5*, AT5G11260; *STOMAGEN*, AT4G12970; *SPCH*, AT5G53210; *COP1*, AT2G32950, and *HYH*, AT3G17609.

### Vector construction and plant transformation

To construct *HY5pro:HY5-YFP*, *HY5* genomic fragment (756 bp upstream of the ATG of *HY5* and its whole-coding region with introns) was PCR-amplified from genomic DNA using primers CACC TCT AAT GTT AAC GTT GAG ATG G (forward) and AAG GCT TGC ATC AGC ATT AGA A (reverse). The PCR product was cloned into pENTR/D-TOPO (Thermo Fisher Scientific, K240020). The sequence of the pENTR clone was validated by DNA sequencing and recombined with the binary vector containing YFP (pHGY)^64^ by Gateway cloning (Thermo Fisher Scientific, 11791020).

To construct *STOMAGENpro:H2B-mScarlet-I*, a *STOMAGEN* promoter fragment (2000 bp upstream of the ATG) was amplified from genomic DNA using primers CCG CGG CCG CCC CCT TCA CCA TAG AAA AGA TTT GCT TCC TAA ACA ATA ATG GTG AAA A (forward) and TTC TCT GCT CTC GGC GCC ATT CTC TAC TTC TTC TTC TTC TTG CTC TAA TTC T (reverse). Separately, the genomic fragment of *H2B* was cloned into pENTR/D-TOPO using primers CACC ATG GCG CCG AGA GCA GAG (forward) and AGA GCT TGT GAA TTT GGT AAC AGC CTT G (reverse) and the cDNA of *mScarlet-I* was fused with *H2B* by HiFi DNA Assembly (New England Biolabs, E2621). The *STOMAGEN* promoter-containing PCR product was then cloned into *pENTR-H2B-mScarlet-I* using the same method. The *pENTR-STOMAGENpro:H2B-mScarlet-I* clone was recombined with the binary vector pGWB401^65^ using Gateway cloning.

To construct the *STOMAGENpro:GUS* reporter, the 500 -bp sequence, upstream to the ATG of *STOMAGEN*, was PCR-amplified using primers CACC GTT CAA AGA GGA GCA AAT CAT (forward) and TCT CTA CTT CTT CTT CTT CTT GCT C (reverse primer). The PCR product was cloned into pENTR/D-TOPO (Thermo Fisher Scientific, K240020). The sequence of the pENTR clone was validated by DNA sequencing and recombined with the binary vector containing GUS (pBGGUS)^64^ by Gateway cloning (Thermo Fisher Scientific, 11791020). To construct the *mSTOMAGENpro:GUS* reporter, site-directed mutagenesis was performed using the above pENTR-*STOMAGENpro* as a template, CCT CTG TAT TTT CAA ACT CTT ATC TCT TGC AGT GCA CAA ACC TCA CCA TTA GAT GAT AAG and CTT ATC ATC TAA TGG TGA GGT TTG TGC ACT GCA AGA GAT AAG AGT TTG AAA ATA CAG AGG as forward and reverse primers, to mutate the Z-box motif from CACGTA to CTGCAA. The mutagenized pENTR plasmid was then recombined into pGGUS, as described above.

To construct *LHCA6Ppro:HY5-YFP*, a *LHCA6* promoter fragment (391 bp upstream of the ATG) was amplified from genomic DNA using primers CCG CGG CCG CCC CCT TCA CCC GTT CGC CGG AGT AAG AGA TTT G (forward) and AGC TAG TCG CTT GTT CCT GCA TCT TTG ATT CGT GGG GAG ATG AAA ACG (reverse). The PCR product was cloned into *pENTR-HY5* using HiFi DNA Assembly. The *pENTR-LHCA6pro:HY5* clone was recombined with the binary vector pHGY^64^ using Gateway cloning.

For the construction of *STOMAGEN-OX*, a *STOMAGEN* genomic fragment (From ATG to stop codon) was amplified from genomic DNA using primers CACC ATG AAG CAT GAA ATG ATG AAC ATC AAG CCA AGA TG (forward) and TTA TCT ATG ACA AAC ACA TCT ATA ATG ATA AGC ACT GTT GAT AGG (reverse). The PCR product was cloned into pENTR/D-TOPO. The sequence of the pENTR clone was validated by DNA sequencing and recombined with the binary vector containing 35S promoter (pH35GS)^64^ by Gateway cloning.

The binary vectors were transformed into WT or *hy5-215* using the floral-dip method^66^ and transgenic plants were selected on ½ MS agar plates containing the relevant antibiotics.

For constructing *MBP-HY5* bacterial expression vector, *HY5* CDS fragment was first PCR-amplified from cDNA using primers CACC ATG CAG GAA CAA GCG ACT A (forward) and TCA AAG GCT TGC ATC AGC ATT AG (reverse). The PCR product was cloned into pENTR/D-TOPO. The resulting pENTR clone was recombined with the expression vector pETG-40A, which contains the maltose-binding protein (MBP) by Gateway cloning. The empty expression vector and the one contains *HY5* were introduced into Rosetta^TM^ DE3 *E. coli* (Sigma-Aldrich, 70954) for protein expression and purification.

### Analysis of transcriptional and translational reporters and the stomatal phenotype of mutants

For confocal microscopy, fluorescence images were captured on an Olympus FV3000 microscope or a Leica SP8 equipped with a Hybrid detector and were processed with ImageJ (National Institutes of Health). Cell outlines were visualized with propidium iodide (Thermo Fisher Scientific, P3566; 0.1 mg/ml).

For quantitative analyses of early stomatal lineage cell populations, meristemoids and guard mother cells (M + GMC) were identified as cells that are either triangular or round in shape and with an average size less than 50 µm^2^. On the other hand, stomatal lineage ground cells (SLGCs) are defined as the larger cells that are adjacent to meristemoids or GMCs, with an average size < 100 µm^2^, and without visible lobe formation. Quantification of nuclei was performed on ImageJ using the Cell Counter plug-in. For imaging the mesophyll, the epidermis of cotyledons was peeled off before imaging to expose the inner tissue and remove signals from the epidermis.

For quantification of stomatal phenotypes, seedlings were first cleared in 7:1 ethanol:acetic acid solution and washed with 70% ethanol. The samples were submerged with chloral hydrate solution (chloral hydrate: water: glycerol, 8:2:1) for 4 h and mounted in the same medium. For a given genotype, differential contrast interference (DIC) images of the abaxial epidermis of cotyledons were captured at ×20 on a Zeiss Axio Imager M2 equipped with a digital CMOS (Hamamatsu, Orca Flash4.0 v2) or a CCD (Zeiss, Axiocam 506) camera (field of view of the image: 0.441 or 0.313 mm^−2^, respectively). Quantification of cells was carried out using ImageJ with the Cell Counter plug-in.

### RT-qPCR analyses

Seedlings at the indicated stage were harvested and flash-frozen in liquid nitrogen, and the total RNA of plants was purified using RNeasy Plant Mini Kit with DNaseI digestion (Qiagen, 74904). Five hundred ng of the RNA was used in reverse transcription using the iScript^TM^ cDNA synthesis kit (Bio-Rad, 1708890). Quantitative PCR were performed with gene-specific primers (Supplementary Table 1) and the LUNA^®^ Universal qPCR Master Mix (New England Biolabs, M3003X) on a CFX96 Real-Time PCR detection system (Bio-Rad). Relative expression of target genes was derived from their target signals normalized against *ACTIN2* or *PP2A* using the Δ^CT^ method.

### Histochemical GUS assays

For *GUS* staining of seedlings from the dark-to-light assay, seedlings grown at the indicated conditions were fixed in 90% acetone at 4 °C overnight. Staining solution [50 mM NA_2_HPO_4_, 50 mM NaHPO_4_, 0.5 mM K_3_Fe(CN)_6_, 0.5 mM K_4_Fe(CN)_6_, 2 mM X-Gluc (DMF)] was then vacuum infiltrated into the seedlings for 25 min, and the seedlings were left for staining in darkness at 37 °C for 1.5 h. The seedlings were later washed serially with 90, 80 and 70% ethanol and were imaged using a wide-field microscope (Leica) equipped with a Nikon DS-Ri1 camera (imaged were captured at ×5).

For GUS staining with light-grown seedlings (Fig. 3h), vacuum-infiltration of the staining solution was carried out for 5 min but the seedlings were left in the solution at 37 °C overnight before imaging.

### Chromatin immunoprecipitation (ChIP) assays

Four-day-old seedlings of *HY5pro:HY5-YFP* in *hy5-1* (in Ler background) and Ler were grown at 22 °C in the dark and transferred to light incubators for 4 h or not before harvested. ChIPs were carried out based on our established MOBE-ChIP method^36,41,67,68^. Briefly, around 12 g of samples were harvested and divided into small aliquots. Chromatin fragmentation was carried out on a Bioruptor^®^ sonicator (Diagenode) with the following settings: 12 high-intensity cycles of 30 s “on” and 30 s “off” at 4 **°**C. Immunoprecipitation was carried out at 4 **°**C overnight on a rotating platform using GFP-TRAP (ChromoTek, gtma-10). Reverse cross-linking of the immunoprecipitated complex was carried out at 65 **°**C for 7 h and purified by the ChIP DNA Clean & Concentrator (Zymo, D5201). Subsequent quantitative PCR reactions were performed with LUNA^®^ Universal qPCR Master Mix (New England Biolabs, M3003X) on a CFX96 Real-Time PCR detection system (Bio-Rad) using primers specific to the promoter of *STOMAGEN* or other specified regions (Supplementary Table 1) (IR1^69^). Signals from the ChIPed DNA were normalized to their input DNA.

### Recombinant protein expression, electrophoretic mobility shift assays (EMSAs), and in vitro DNA pull-down assays

For recombinant MBP-HY5 expression, transformed *E. coli* was induced with 0.5 mM IPTG (Thermo Fisher Scientific, 15529019) at 37 **°**C for 4 h. The recombinant protein was purified with amylose resin (New England Biolabs, E8021). For EMSA, a 20-µl reaction mixture containing binding buffer, 20-fmol biotin-labeled *STOMAGEN* probe (P2, Supplementary Table 2), varying amounts of unlabeled probes as competitors, and recombinant proteins (~ 1 µg) was set on ice for 1 h. DNA–protein complexes were resolved on a 5% polyacrylamide gel and transferred to a charged Hybond-N membrane (GE Healthcare). The membrane was cross-linked by UV and probed with streptavidin-AP (Thermo Fisher Scientific, 21130; 1:5000 dilution), and the signal was developed using chemiluminescent substrate (Thermo Fisher Scientific, 34075).

For in vitro DNA pull-down assay, 10 pmol biotin-labeled probes (Supplementary Table 2) were first incubated with 10 µl Dynabeads^TM^ M-280 Streptavidin magnetic beads (Thermo Fisher Scientific) for 15 min at room temperature in the 1 × B&W buffer [5 mM Tris-HCl (pH 7.4), 0.5 mM EDTA, 1 M NaCl]. The probes-bound beads were then incubated with the same amount of MBP or MBP-HY5 protein (~ 100 ng) in the 100 µl binding buffer [10 mM Tris·HCl (pH 7.5), 50 mM KCl, 5 mM MgCl_2_, 2.5% glycerol, 0.05% Nonidet P-40] supplemented with 5 µg of fragmented salmon sperm DNA and 10 µg BSA for 1 h at 4 °C. The precipitates were eluted in the SDS loading buffer and subjected to western blot analysis using an anti-MBP monoclonal antibody (NEB, E8032S, 1:10,000 dilution), followed by anti-mouse IgG-HRP (Sigma, A4416, 1:10,000 dilution).

### Western blotting

Total proteins were extracted from seedlings using 1× Laemmli sample buffer (Bio-Rad, 1610737) and immunoblotting was carried out with an HRP-conjugated anti-GFP antibody (Miltenyi Biotec, 130-091-83, 1:3000 dilution) or a Rabbit monoclonal anti-GFP antibody (Cell Signaling Technology, 2956, 1:1000 dilution) followed by anti-rabbit IgG-HRP (Cell Signaling Technology, 7074, 1:3000 dilution). Either cross-reacting bands or proteins stained by Ponceaus S (Sigma-Aldrich, P3504; 0.1% w/v in 5% acetic acid) were used as loading controls. Chemiluminescent substrate (Thermo Fisher Scientific, 34075) was used for signal detection.

### Reporting summary

Further information on research design is available in the Nature Research Reporting Summary linked to this article.

## Supplementary information

Supplementary Information

Reporting Summary

## Data Availability

All relevant data are available from the authors.  Source data are provided with this paper.

## Source data

Source data file
