# Supplementary material for: Light regulates stomatal development by modulating paracrine signaling from inner tissues
Source: Nat Commun. 2021 Jun 7;12:3403. doi: 10.1038/s41467-021-23728-2 (PMC8184810; doi:10.1038/s41467-021-23728-2)
Supplement: Supplementary file 1 — Supplementary Information [file 41467_2021_23728_MOESM1_ESM.pdf]

Supplementary Information

Light regulates stomatal development by modulating paracrine signaling from inner tissues

Shenqi Wang[†,‡], Zimin Zhou[†], Rini Rahiman, Grace Sheen Yee Lee, Yuan Kai Yeo, Xin Yang and On Sun Lau*

Department of Biological Sciences, National University of Singapore, 14 Science Drive 4, Singapore 117543, Singapore
[†]These authors contributed equally to this work
[‡]Present address: Department of Molecular, Cellular and Developmental Biology, Yale University, New Haven, CT 06520, USA
*Correspondence should be addressed to O.S.L. (email: onsunlau@nus.edu.sg)

List of supplementary figures and tables

Supplementary Fig. 1. Effect of light intensity on stomatal development in *hyh-1* and the double mutants *hy5-51 hyh-1* and *hyh-1 cop1-6*.

Supplementary Fig. 2. Effect of light intensity on stomatal development in *cop1-6* and the double mutant *hy5-215 cop1-6*.

Supplementary Fig. 3. Gene expression analysis of two direct targets of SPCH in WT and *hy5* mutant during dark to light transition.

Supplementary Fig. 4. Analysis of a MAPK-insensitive translational reporter of *SPCH* in *hy5* mutant.

Supplementary Fig. 5. Genetic interaction between *HY5* and *SPCH*.

Supplementary Fig. 6. Expression analyses of the *STOMAGEN-VENUS* line in WT and *hy5*.

Supplementary Fig. 7. *GUS* reporter analyses of the promoter activity of *STOMAGEN* in wild-type and *hy5* mutant.

Supplementary Fig. 8. Expression pattern of a translational reporter of *HY5* in mesophyll and the epidermis.

Supplementary Fig. 9. Purification of recombinant HY5 proteins, additional EMSA assay of HY5 and expression dynamics of *HY5pro:HY5-YFP* during dark to light transition

Supplementary Fig. 10. Density of stomata in WT, *stomagen* and *hy5* mutants.

Supplementary Fig. 11. Confocal analyses of the mesophyll layer of *HY5pro:HY5-YFP* under low and high light.

Supplementary Fig. 12. Phenotypic analyses of hypocotyl growth of WT, *hy5-215*, *HY5-OX* and *HY5pro:HY5-YFP hy5-215*.

Supplementary Table 1. List of primers for RT-qPCR and ChIP-qPCR

Supplementary Table 2. List of probes for EMSAs and DNA pull-down assays

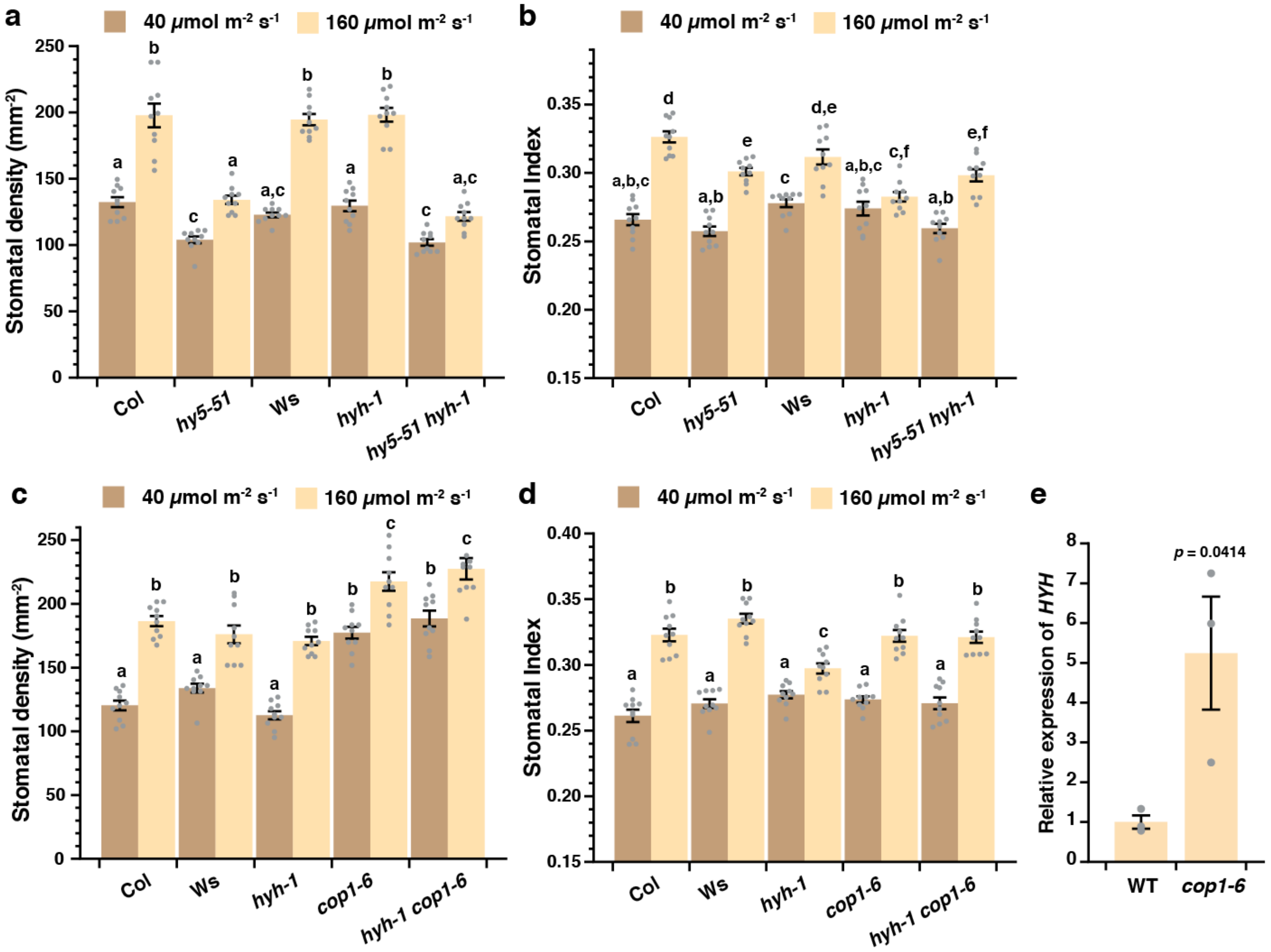

Supplementary Fig. 1. Effect of light intensity on stomatal development in *hyh-1* and the double mutants *hy5-51 hyh-1* and *hyh-1 cop1-6*. (**a**, **b**) Stomatal densities (a) and indices (b) of the abaxial cotyledons of 10-dpg seedlings of Col, *hy5-51*, Ws, *hyh1* and *hy5-51 hyh-1* grown under low (40 µmol m^{-2} s^{-1}) and high (160 µmol m^{-2} s^{-1}) light intensity. *hy5-51* and *hyh-1* are in Col and Ws ecotypes, respectively. (**c**, **d**) Stomatal densities (c) and indices (d) of the abaxial cotyledons of 10-dpg seedlings of Col, Ws, *hyh1*, *cop1-6* and *hyh-1 cop1-6* grown under low and high light (as above). (**e**) Gene expression analysis of *HYH* in WT and *cop1-6* by RT-qPCR. RNA was extracted from 3-day-old seedlings grown under 100 µmol m^{-2} s^{-1} of light. Values are mean +/- SEM, n = 10 independent cotyledons (a-d) or 3 biological replicates (e). Two-way ANOVA with Tukey's multiple comparisons test (a-d) or two-tailed Student's t-test (e), $p<0.05$.

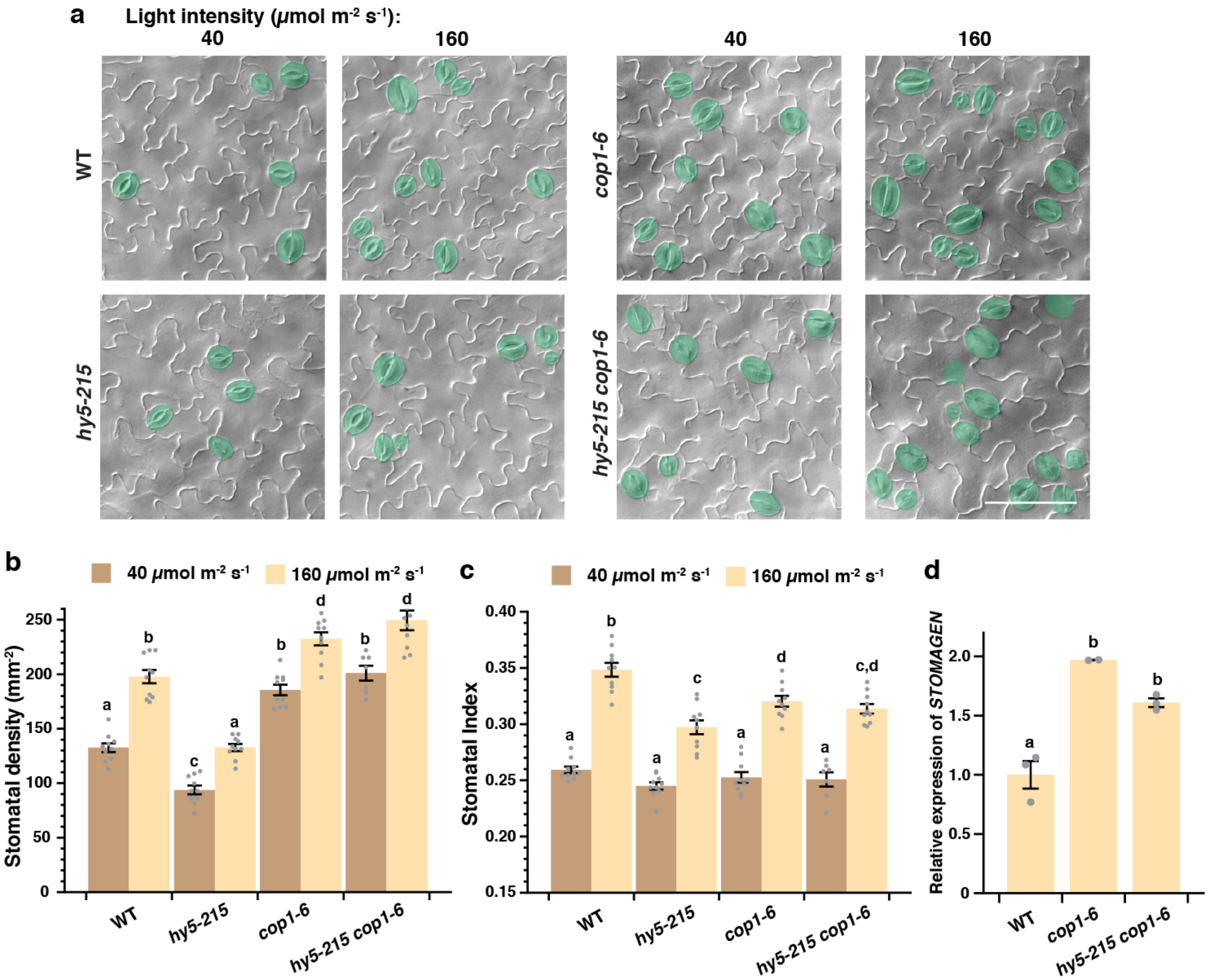

Supplementary Fig. 2. Effect of light intensity on stomatal development in *cop1-6* and the double mutant *hy5-215 cop1-6*. (**a**-**c**) Arabidopsis seedlings of WT, *hy5-215*, *cop1-6* and *hy5-215 cop1-6* were grown for 10 days at 22°C under two distinct light intensities (40 and 160 μmol m^{-2} s^{-1}). Representative images (a), stomatal densities (b) and stomatal indices (c) of the abaxial cotyledons are shown. Stomata are pseudo-colored in green (a). Scale bar, 80 μm. (**d**) Gene expression analysis of *STOMAGEN* in the indicated genotypes. RNA was extracted from 3-day-old seedlings grown under 100 μmol m^{-2} s^{-1} of light. Values are mean +/- SEM, n = 10 independent cotyledons (b-c), 3 (d, WT & *hy5-215 cop1-6*) or 2 (d, *cop1-6*) biological replicates. Two-way (b, c) or one-way (d) ANOVA with Tukey's multiple comparisons test, $p<0.05$.

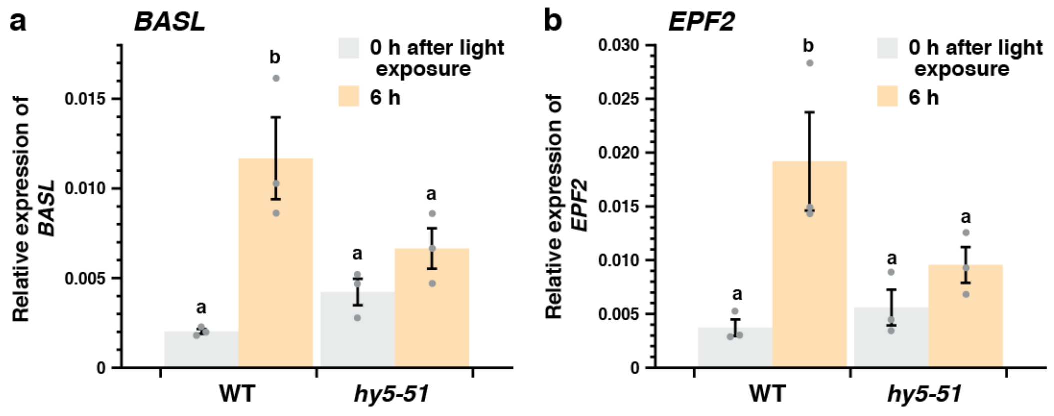

Supplementary Fig. 3. Gene expression analysis of two direct targets of SPCH in WT and *hy5* mutant during dark to light transition. Seedlings of WT and *hy5-51* were grown in darkness for 4 days and were exposed to light for 0 and 6 h before RNA extraction. RT-qPCR was performed to monitor the expression of *BASL* (a) and *EPF2* (b). Signals are normalized with *ACTIN2*. Values are mean +/- SEM, n = 3 biological replicates. Two-way ANOVA with post-hoc Tukey HSD, $p<0.05$.

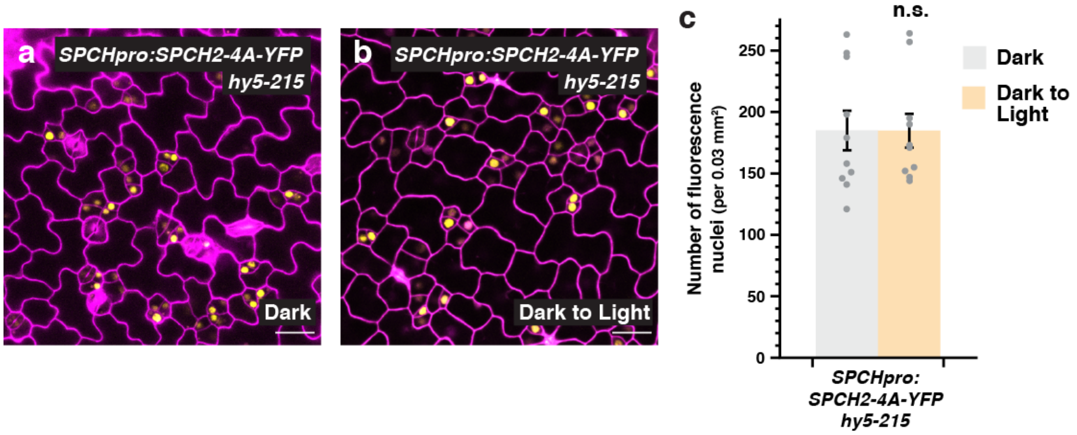

Supplementary Fig. 4. Analysis of a MAPK-insensitive translational reporter of *SPCH* in *hy5* mutant. Confocal images of 3-day-old abaxial cotyledons of *SPCHpro:SPCH2-4A-YFP* in *hy5-215* grown in darkness (a) or exposed to light (b) (see Fig. 2). The numbers of YFP-expressing cells (yellow) are quantified (c). Values are mean +/- SEM, n = 10 independent cotyledons. Two-tailed Student’s t-test, n.s., not significant. Cell outlines were visualized with propidium iodide (magenta). Scale bar, 20 μm.

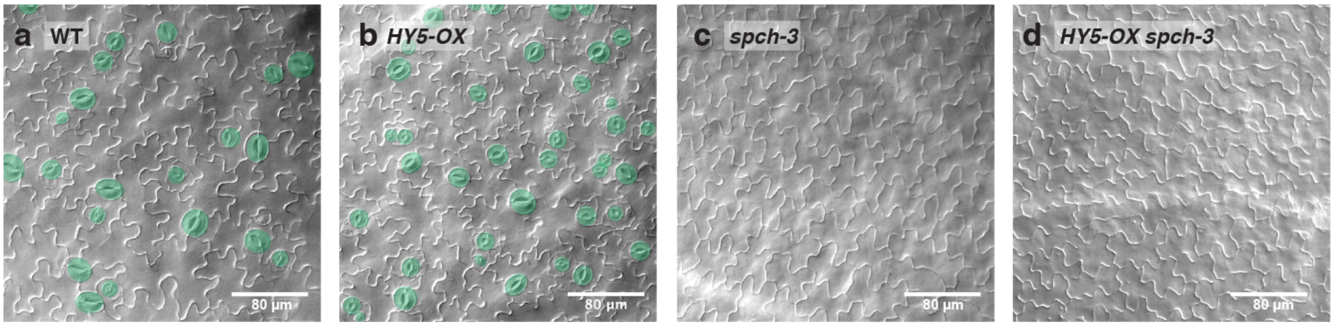

Supplementary Fig. 5. Genetic interaction between *HY5* and *SPCH*. Representative images of the epidermis of the adaxial cotyledons of WT (a), *HY5-OX* (b), *spch-3* (c) and the *HY5-OX spch-3* double mutants (d) are shown. Seedlings were grown for 10 days under light. Scale bar, 80 μm. Three cotyledons from each genotype were examined with similar results.

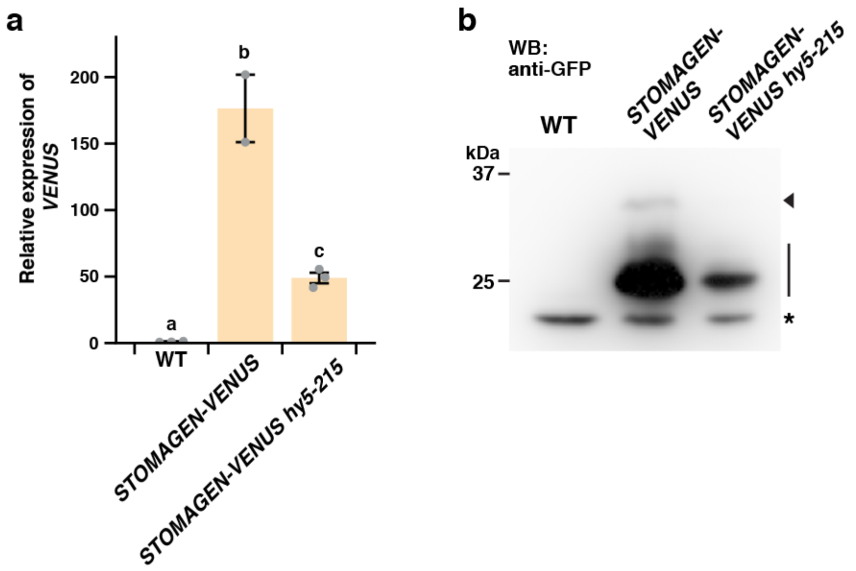

Supplementary Fig. 6. Expression analyses of the *STOMAGEN-VENUS* line in WT and *hy5*. (**a**) Expression analysis of the *VENUS* transcripts of 3-day-old light-grown seedlings of WT, *STOMAGENpro:STOMAGEN-VENUS* and *STOMAGENpro:STOMAGEN-VENUS* in *hy5-215* by RT-qPCR. RNA was extracted from 3-day-old seedlings grown under 100 μmol m^{-2} s^{-1} of light. Values are mean +/- SEM, n = 3 (WT & *STOMAGENpro:STOMAGEN-VENUS hy5-215*) or 2 (*STOMAGENpro:STOMAGEN-VENUS*) biological replicates. One-way ANOVA with Tukey's multiple comparisons test, $p<0.05$. (**b**) Western blot analysis of a translational reporter of *STOMAGEN*. Total proteins were extracted from the above plant lines. Protein extracts were probed with an anti-GFP antibody. Arrowhead: full-length STOMAGEN-VENUS, Line: degraded products of STOMAGEN-VENUS, Asterisk: non-specific band. The experiment was repeated two times with similar results.

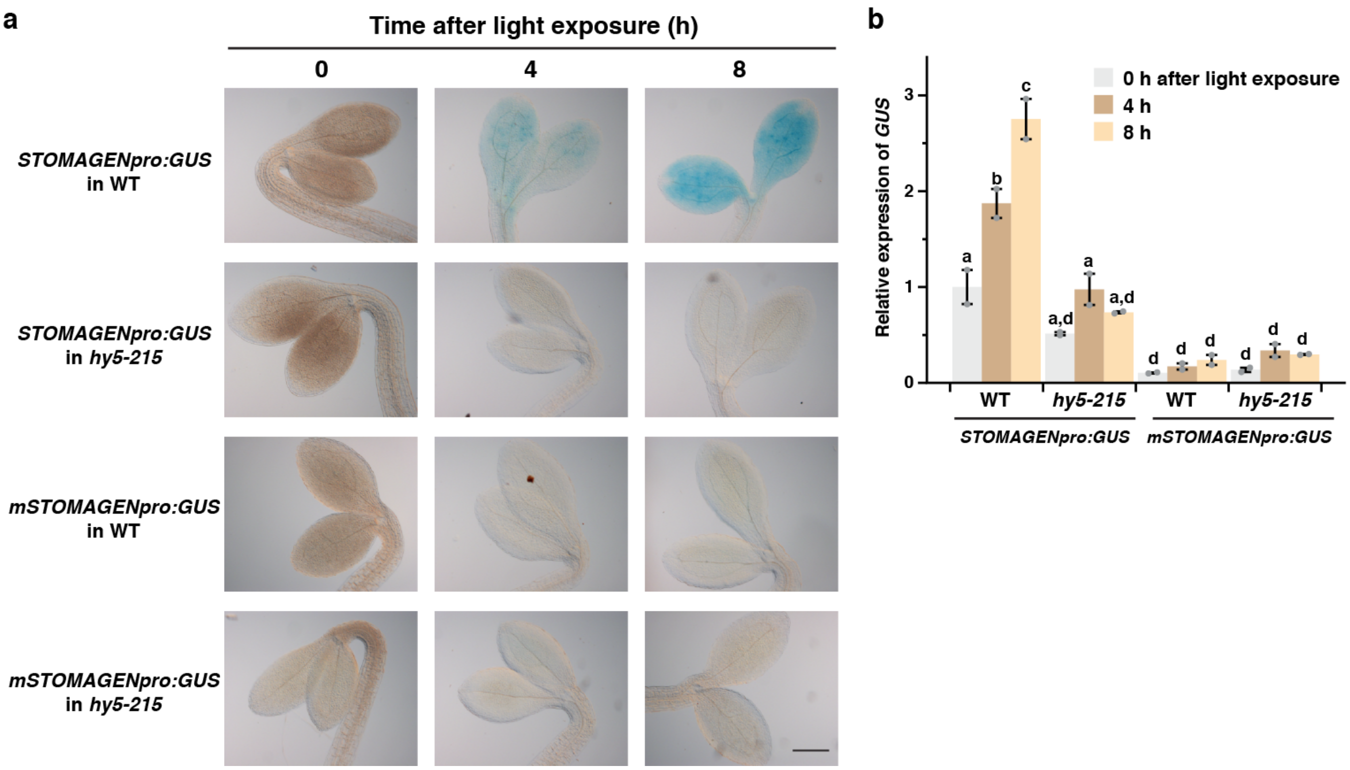

Supplementary Fig. 7. *GUS* reporter analyses of the promoter activity of *STOMAGEN* in wild-type and *hy5* mutant. (**a**) Histochemical GUS staining of the indicated *GUS* reporters in wild-type (WT) and in *hy5-215*. *STOMAGENpro:GUS* refers to a *GUS* reporter gene driven by the *STOMAGEN* promoter, whereas *mSTOMAGENpro:GUS* denotes the same *GUS* reporter driven by a Z-box mutated version of the *STOMAGEN* promoter. Seedlings were grown in darkness for 3 days and were exposed to light for 0, 4 and 8 h before harvest. Scale bar, 0.4 mm. (**b**) Gene expression analyses of the *GUS* transcript of the *GUS* reporter lines in (a) by RT-qPCR. Four dpg seedlings were grown in darkness and were exposed to light for 0, 4 and 8 h before harvest and RNA extraction. Values are mean +/- SEM, n = 2 biological replicates (each assayed with 3 technical replicates). Two-way ANOVA with Tukey's multiple comparisons test, $p<0.05$.

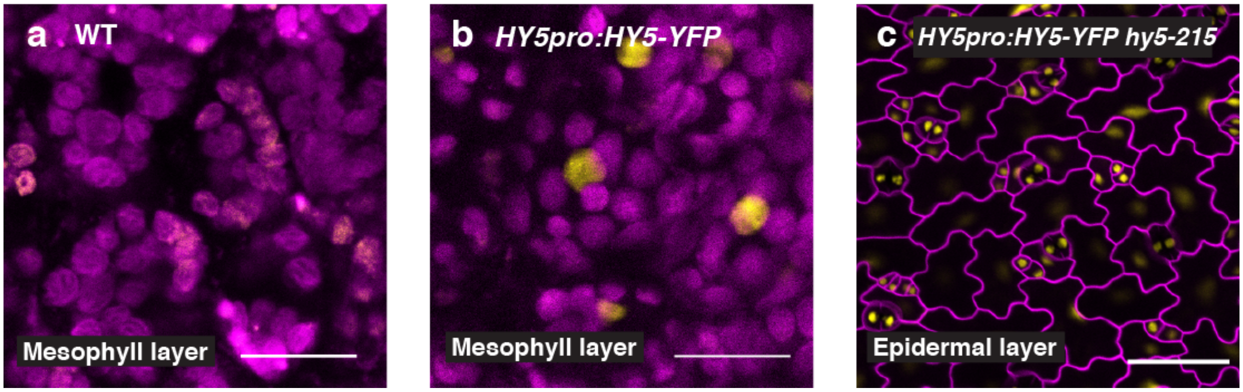

d

Supplementary Fig. 8. Expression pattern of a translational reporter of *HY5* in mesophyll and the epidermis. (**a**, **b**) Confocal analysis of the mesophyll layer of 3-day-old abaxial cotyledons of WT (a) and *HY5pro:HY5-YFP* (b) grown under standard conditions. (**c**) Confocal analysis of the epidermis of 3-day-old abaxial cotyledons of *HY5pro:HY5-YFP*. Yellow: YFP signals (a-c), Magenta: Chloroplasts' auto-fluorescence (a, b), cell outline (c). Scale bar, 20 μm (a, b), 50 μm (c). Three cotyledons of the above genotypes were examined with similar results.

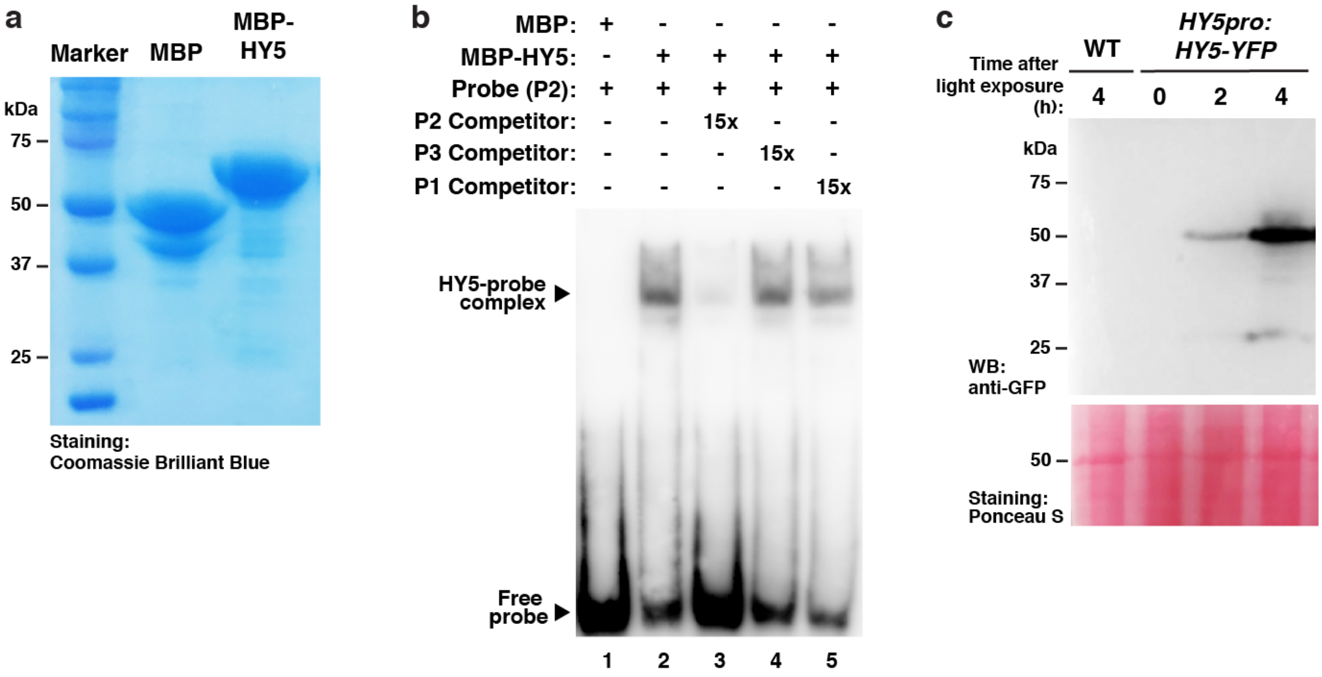

Supplementary Fig. 9. Purification of recombinant HY5 proteins, additional EMSA assay of HY5 and expression dynamics of *HY5pro:HY5-YFP* during dark to light transition. (**a**) Recombinant Maltose-Binding Protein (MBP) and MBP-tagged HY5 were expressed and purified from *E. coli* (see Methods). Purified proteins were analyzed on SDS-PAGE and stained with Coomassie Brilliant Blue. (**b**) EMSA analysis showing the effectiveness of different regions of the *STOMAGEN* promoter in interfering the binding of HY5 to the P2 fragment of the *STOMAGEN* promoter. Recombinant MBP-HY5 was assayed for binding with the biotin-labelled P2 probe (lane 1 and 2; refer to Fig. 3d, e). Un-labelled competitors (P2, P1 and P3) (see Fig. 3d) was used to compete against this binding (lane 3-5). (**c**) Expression of *HY5pro:HY5-YFP* during dark to light transition. Seedlings of WT and *HY5pro:HY5-YFP* were grown in darkness for 4 days and were exposed to light for 0, 2 and 4 h before harvest. Total proteins were extracted and western blot analysis was carried out using an anti-GFP antibody (Cell Signaling Technology, #2956). Ponceau S staining of the total blotted proteins was served as loading controls. The SDS-PAGE analysis of the purified proteins in (a) was carried out one time. The experiments in (b) and (c) were repeated two times with similar results.

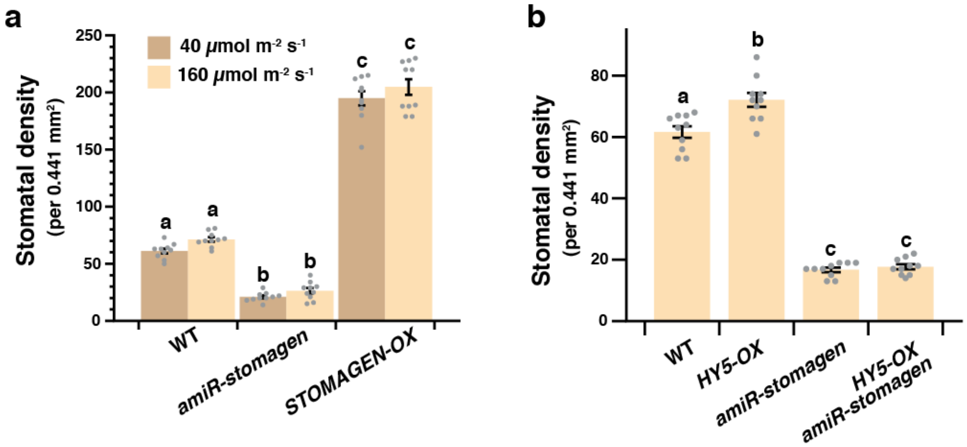

Supplementary Fig. 10. Density of stomata in WT, *stomagen* and *hy5* mutants. (**a**) Quantification of the density of stomata in 10-day-old wild-type (WT), *amiR-stomagen* and *STOMAGEN-OX* abaxial cotyledons grown under low or high light (40 and 160 μmol m^{-2} s^{-1}, respectively) using the same plant samples as in Fig. 5g. (**b**) Quantification of the density of stomata in wild-type (WT), *HY5-OX*, *amiR-stomagen* and *HY5-OX amiR-stomagen* abaxial cotyledons grown for 10 days using the same plant samples as in Fig. 5l. Values are mean +/- SEM, n = 10 independent cotyledons. Two-way (a) and one-way (b) ANOVA with post-hoc Tukey HSD, $p<0.01$.

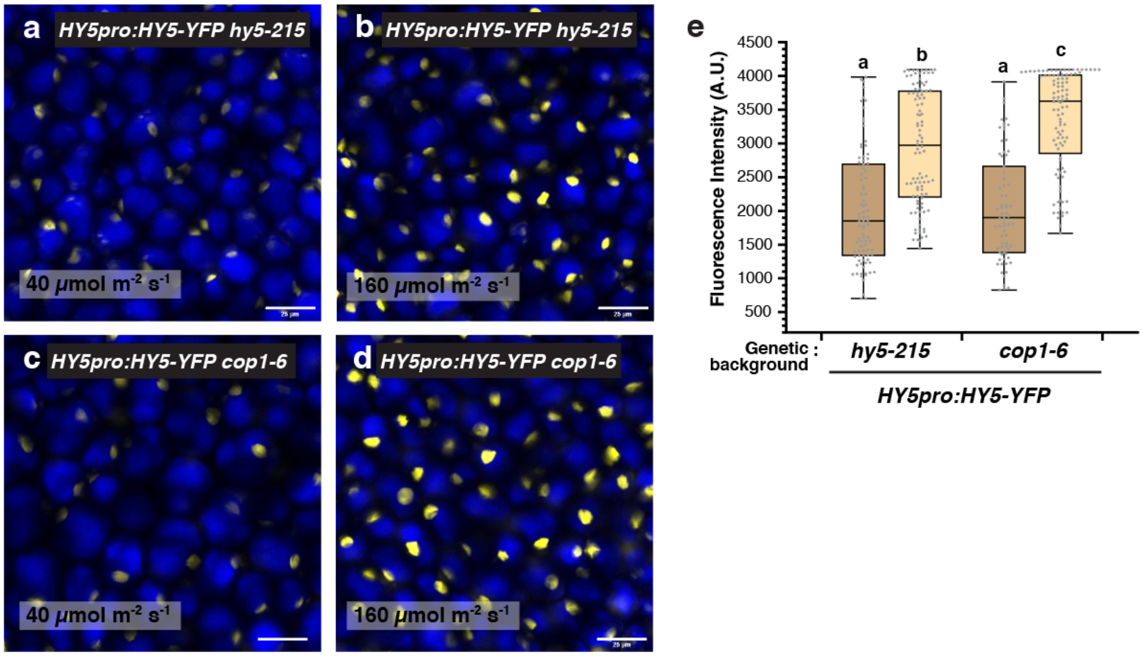

Supplementary Fig. 11. Confocal analyses of the mesophyll layer of *HY5pro:HY5-YFP* under low and high light. (**a**-**d**) *HY5pro:HY5-YFP* in either *hy5-215* (a, b) or *cop1-6* (c, d) were grown for 3 days under low or high light (40 and 160 μmol m^{-2} s^{-1}). Expression of the *HY5pro:HY5-YFP* in the mesophyll layer were imaged by confocal microscopy. Yellow: YFP signals, Blue: Chloroplasts' auto-fluorescence. Scale bar, 25 μm. (**e**) Quantification of the intensity of the YFP signals of the above samples. Center line of box plots, median; Box limits, first and third quartiles; Whiskers, minimum and maximum values. n = 70 nuclei. Two-way ANOVA with Tukey's multiple comparisons test, $p<0.01$.

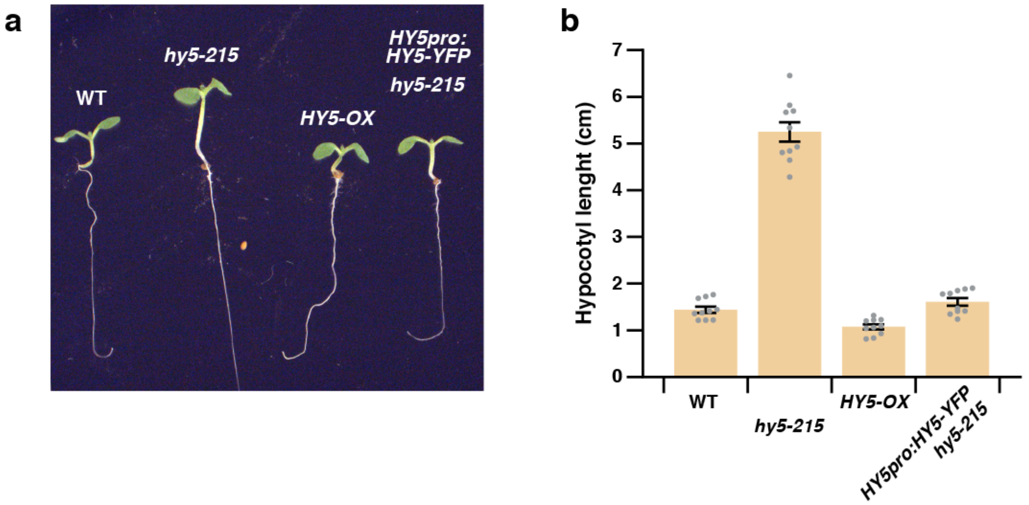

Supplementary Fig. 12. Phenotypic analyses of hypocotyl growth of WT, *hy5-215*, *HY5-OX* and *HY5pro:HY5-YFP hy5-215*. (**a**) Image of 7-day-old seedlings of WT, *hy5-215*, *HY5-OX* and *HY5pro:HY5-YFP hy5-215* grown under low light intensity (40 µmol m^{-2} s^{-1}). (**b**) Hypocotyl length measurements of the seedlings shown in (a). Values are mean +/- SEM, n = 10 seedlings.

Supplementary Table 1. List of primers for RT-qPCR and ChIP-qPCR

Primers for RT-qPCR (all written from 5' to 3')

Gene name	AGI code	Forward primer	Reverse primer	Amplicon size (bp)
ACTIN2	AT3G18780	AAGCTGGGGTTTTATGAATGG	TTGTCACACACAAGTGCATCAT	118
PP2A	AT4G12970	CAAGTGAACCAGGTTATTGGGA	ATAGCCAGACGTACTCTCCAG	101
STOMAGEN	AT4G12970	TAGGGTCGACAGCACCAACTTGTAC	TCATTTCCTTCGACTGGAACTTGCT	91
BASL	AT5G60880	CCTCTAGACGGAGATGAAGATGG	CCTGGTGGGCTTAGGCTGAG	143
EPF2	AT1G34245	CGCGTGTTCTTTGGTCGTTAAC	CCTTCTTGTGGTGCGTTTGAG	113
HYH	AT3G17609	CAATGGGAACTCGAGTTCGTCTTCT	TGATCCAGCTGCTTCCATGTCAGGA	100
VENUS	--	GTAAACGGCCACAAGTTCAGCGTG	TTGCCGGTGGTGCAGATCAGCTT	92
GUS	--	GTGCTGTGCCTGAACCGTTATTAC	GGCTAACGTATCCACGCCGTATTC	150

Primers for ChIP-qPCR (all written from 5' to 3')

Name in study	Distance from start codon of ***STOMAGEN*** (bp)	Forward primer	Reverse primer	Amplicon size (bp)
P1	-1	CAAACAGGCAAACTTATCTCTCTC	TCTCTACTTCTTCTTCTTCTTGCT	97
P2	-105	ATCTTGTGGGTTAAAAGTGAAAACT	TTTTGTCTTCTACTTATCATCTAATGGTG	100
P3	-215	AACATACTAGAAGAGTTAAATATATGTTTG	AGAATAAAAAACATTTAACTCTTCTCTTT	100
3'	+2250	TTCAGGCGTTTTTCATGTTC	CGTATTGAACTAAGATGGATTTAAGC	120

Supplementary Table 2. List of probes for EMSAs and DNA pull-down assays

Name in study	Distance from start codon of ***STOMAGEN*** (bp)	**Sequence** (from 5' to 3')	Size (bp)
P1	-22	TCTCTCTCCACAACATTTGGTCCATACGATAAAGCAAGAAGAAGAATTAAGAATTAGAGC	60
P2	-119	TCCTCTGTATTTTCAAACTCTTATCTCTACGTGTGCACAAACCTCACCATTAGATGATAA	60
P3	-235	ATATGTTTGACCGAAGAAAAATAGAGTTAAATATGTTGTGCTAAAAGGATAAAGAGAAGA	60
mP2	-119	TCCTCTGTATTTTCAAACTCTTATCTCAAAAAATGCACAAACCTCACCATTAGATGATAA	60
