# Supplementary material for: Light regulates stomatal development by modulating paracrine signaling from inner tissues
Source: Nat Commun. 2021 Jun 7;12:3403. doi: 10.1038/s41467-021-23728-2 (PMC8184810; doi:10.1038/s41467-021-23728-2)
Supplement: Supplementary file 2 — Reporting Summary [file 41467_2021_23728_MOESM2_ESM.pdf]

nature research

Corresponding author(s): On Sun Lau

Last updated by author(s): May 5, 2021

Reporting Summary

Nature Research wishes to improve the reproducibility of the work that we publish. This form provides structure for consistency and transparency in reporting. For further information on Nature Research policies, see Authors & Referees and the Editorial Policy Checklist .

Statistics

For all statistical analyses, confirm that the following items are present in the figure legend, table legend, main text, or Methods section.

n/a	Confirmed	
☐	☒	The exact sample size (*n*) for each experimental group/condition, given as a discrete number and unit of measurement
☐	☒	A statement on whether measurements were taken from distinct samples or whether the same sample was measured repeatedly
☐	☒	The statistical test(s) used AND whether they are one- or two-sided<br>*Only common tests should be described solely by name; describe more complex techniques in the Methods section.*
☒	☐	A description of all covariates tested
☒	☐	A description of any assumptions or corrections, such as tests of normality and adjustment for multiple comparisons
☐	☒	A full description of the statistical parameters including central tendency (e.g. means) or other basic estimates (e.g. regression coefficient) AND variation (e.g. standard deviation) or associated estimates of uncertainty (e.g. confidence intervals)
☐	☒	For null hypothesis testing, the test statistic (e.g. *F*, *t*, *r*) with confidence intervals, effect sizes, degrees of freedom and *P* value noted<br>*Give P values as exact values whenever suitable.*
☒	☐	For Bayesian analysis, information on the choice of priors and Markov chain Monte Carlo settings
☒	☐	For hierarchical and complex designs, identification of the appropriate level for tests and full reporting of outcomes
☒	☐	Estimates of effect sizes (e.g. Cohen's *d*, Pearson's *r*), indicating how they were calculated

Our web collection on statistics for biologists contains articles on many of the points above.

Software and code

Policy information about availability of computer code

Data collection	Olympus cellSens Dimension and Zeiss Zen Pro
Data analysis	Image analysis: Fiji-ImageJ version 2.1.0/1.53g, with the Cell Counter plug-in<br>Statistical analysis: Microsoft Excel 2018, GraphPad Prism version 6, or an online tool <http://astatsa.com/OneWay_Anova_with_TukeyHSD>

For manuscripts utilizing custom algorithms or software that are central to the research but not yet described in published literature, software must be made available to editors/reviewers. We strongly encourage code deposition in a community repository (e.g. GitHub). See the Nature Research guidelines for submitting code & software for further information.

Data

Policy information about availability of data

All manuscripts must include a data availability statement. This statement should provide the following information, where applicable:

- Accession codes, unique identifiers, or web links for publicly available datasets
- A list of figures that have associated raw data
- A description of any restrictions on data availability

Accession codes of the genes involved were included in the Methods. A Source file containing associated raw data has been included. There is no restriction on data availability.

Field-specific reporting

Please select the one below that is the best fit for your research. If you are not sure, read the appropriate sections before making your selection.

Life sciences study design

All studies must disclose on these points even when the disclosure is negative.

Sample size	Sample size was determined based on general practice in the field. Refer to figure legends and Methods for the sample size number.
Data exclusions	Besides poorly prepared samples and outliers, including the occasional seedlings with stunted growth, no data were excluded from the analyses.
Replication	All attempts of replication were successfully.
Randomization	Plates containing the seedlings were placed in a random order and seedlings are harvested/analyzed in a random manner.
Blinding	We did not use binding but key experiments were examined/counted by other members of the team.

Reporting for specific materials, systems and methods

We require information from authors about some types of materials, experimental systems and methods used in many studies. Here, indicate whether each material, system or method listed is relevant to your study. If you are not sure if a list item applies to your research, read the appropriate section before selecting a response.

Materials & experimental systems

n/a	Involved in the study	
☐	☒	Antibodies
☒	☐	Eukaryotic cell lines
☒	☐	Palaeontology
☒	☐	Animals and other organisms
☒	☐	Human research participants
☒	☐	Clinical data

Methods

n/a	Involved in the study	
☒	☐	ChIP-seq
☒	☐	Flow cytometry
☒	☐	MRI-based neuroimaging

Antibodies

Antibodies used	Commercial antibodies used: GFP-Trap magnetic agarose (Chromotek, gtma-10), anti-GFP-HRP (Miltenyi Biotec, 130-091-833), anti-GFP (Cell Signaling Technology, 2956), anti-MBP monoclonal antibody (NEB, E8032S), anti-mouse IgG-HRP (Sigma, A4416) and anti-rabbit IgG-HRP (Cell Signaling Technology, 7074).
Validation	Please refer to the manufarutrer pages below for the validation statement. Proper controls (un-tagged WT) were used in the relevant experiments.<br>https://www.chromotek.com/nc/products/detail/product-detail/gfp-trap-magnetic-agarose/<br>https://www.miltenyibiotec.com/SG-en/products/macs-flow-cytometry/antibodies/primary-antibodies/anti-gfp-antibodies-gg4-2c2-12-10.html#hrp:100-ul<br>https://www.cellsignal.com/products/primary-antibodies/gfp-d5-1-xp-rabbit-mab/2956<br>https://www.neb.sg/products/e8032-anti-mbp-monoclonal-antibody#Product%20Information<br>https://www.sigmaaldrich.com/catalog/product/sigma/a4416?lang=en&region=SG<br>https://www.cellsignal.com/products/secondary-antibodies/anti-rabbit-igg-hrp-linked-antibody/7074
